# Hydroxyl radical chemistry in advanced oxidation processes: a cross-system mechanistic framework and catalyst design perspectives

**DOI:** 10.1039/d6ra06527a

**Published:** 2026-09-23

**Authors:** Hoang Hien Y., Nguyen Tien Hoang, Nguyen Thi Thy Nga, Bui Dinh Nhi, Minh Thi Thao, Hai Son Truong-Lam, Ha Huu Do

**Affiliations:** a Center for Advanced Chemistry, Institute of Research & Development, Duy Tan University 03 Quang Trung Da Nang City 550000 Vietnam; b Faculty of Environmental and Natural Sciences, Duy Tan University 03 Quang Trung Da Nang City 550000 Vietnam; c The University of Danang, University of Science and Education 459 Ton Duc Thang St. Da Nang 550000 Vietnam nthoang@ued.udn.vn; d Faculty of Applied Science and Engineering, Electric Power University Ha Noi Vietnam; e Faculty of Energy and Sustainable Development, Electric Power University Ha Noi Vietnam; f Faculty of Chemistry, University of Science, Vietnam National University Ho Chi Minh City 70000 Vietnam; g NTT Hi-Tech Institute, Nguyen Tat Thanh University Ho Chi Minh City Vietnam; h Nguyen Tat Thanh University Center for Hi-Tech Development Saigon Hi-Tech Park Ho Chi Minh City Vietnam

## Abstract

Hydroxyl radicals (˙OH) are widely recognized as the most powerful oxidizing species in advanced oxidation processes (AOPs) and play a central role in the degradation of persistent organic contaminants. However, current understanding of ˙OH chemistry remains fragmented because radical generation is typically interpreted within technology-specific frameworks, limiting cross-system mechanistic understanding and catalyst development. This review establishes a unified framework for ˙OH generation across AOPs and demonstrates that diverse technologies can be interpreted through three recurring mechanistic pathways: oxidant activation, ˙OH generation from water through oxidation or dissociation, and metal redox cycling. Furthermore, we introduce the concept of radical convergence, highlighting that reactive oxygen species (ROS), including SO_4_˙^−^, O_2_˙^−^, ^1^O_2_, 
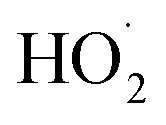
, *etc.*, generated from different oxidants can participate in interconnected ROS reaction networks, in which ˙OH represents an important reactive species under appropriate conditions. Based on this framework, the relative contribution of ˙OH across representative AOPs is systematically compared, together with the relationships among radical generation, radical utilization, competing ROS, and water-matrix effects. A key contribution of this review is the introduction of ˙OH utilization efficiency (*η*_˙OH_) as a conceptual descriptor linking radical generation with effective pollutant oxidation. Building upon this perspective, catalyst design principles are proposed, including interfacial confinement of ˙OH, local pollutant enrichment, pathway optimization, and matrix-tolerant catalyst development. Future opportunities in ˙OH engineering are also discussed, with emphasis on quantitative radical-flux analysis and data-driven, mechanism-guided catalyst discovery. By shifting the focus from technology-specific classifications to ˙OH-centered reaction networks, this review provides a unified mechanistic perspective and mechanism-guided design principles for advancing AOP catalyst development.

## Abstract 2

Hydroxyl radicals (˙OH) are widely recognized as the most powerful oxidizing species in advanced oxidation processes (AOPs) and play a central role in the degradation of persistent organic contaminants.

## Graphical abstract

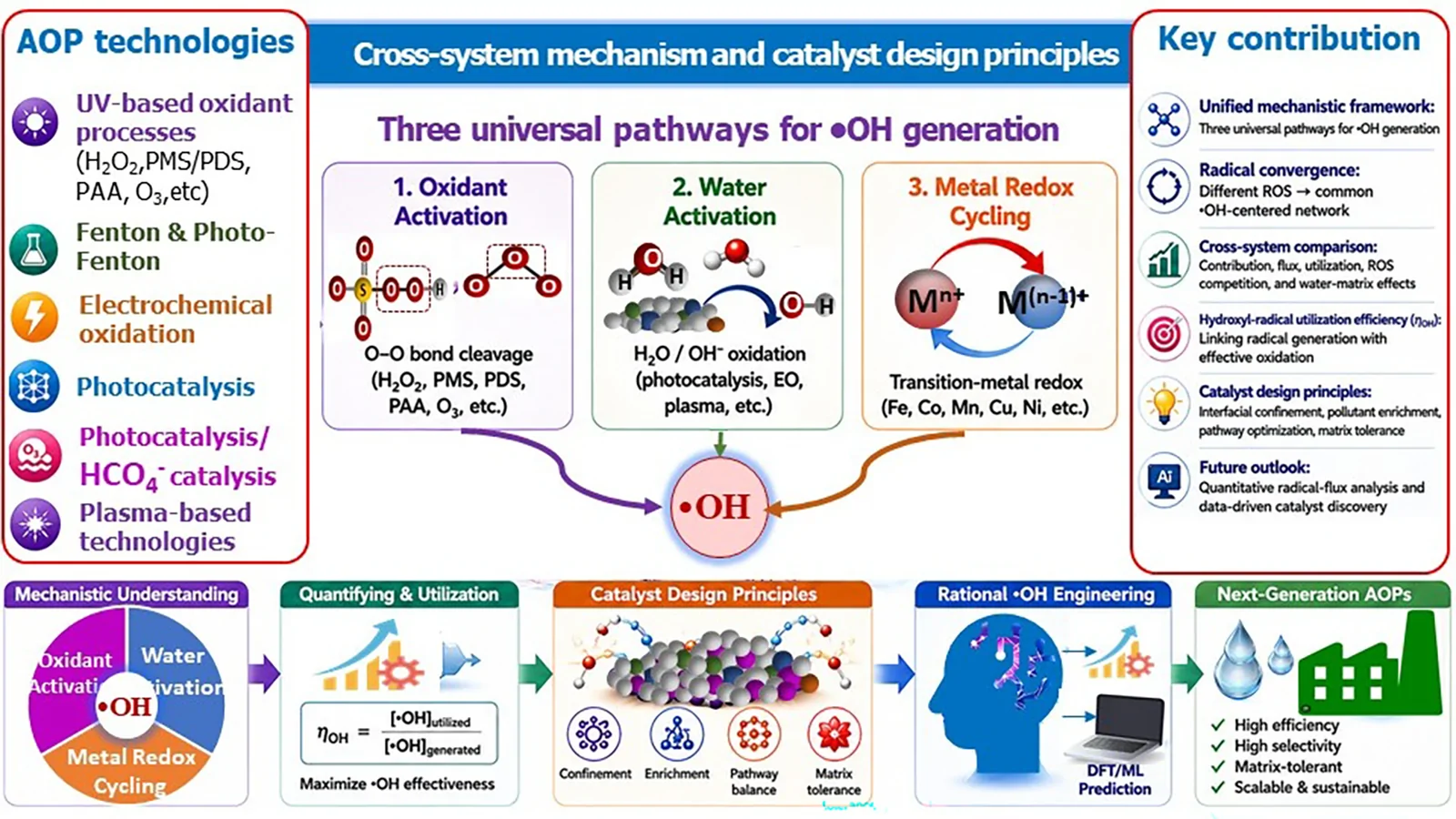

## 1. Introduction

Hydroxyl radicals are the most important reactive species in AOPs owing to their extremely high oxidation potential and broad, nonselective reactivity toward a wide range of refractory contaminants, including pharmaceuticals, dyes, pesticides, and per- and polyfluoroalkyl substances (PFAS).^1–4^ Consequently, ˙OH plays a central role in pollutant degradation and mineralization across diverse AOPs, such as UV-based oxidation systems,^5–7^ Fenton and photo-Fenton processes,^8–10^ lectrochemical oxidation,^11–15^ photocatalysis,^16–20^ catalytic ozonation,^21–23^ and plasma technologies.^24,25^ Beyond aqueous-phase AOPs, ˙OH also contributes to gas-phase oxidation of organic pollutants, including hydrocarbon oxidation in nonthermal plasma systems and photochemical oxidation of atmospheric trace gases.^26,27^ Within aqueous AOPs, despite differences in oxidants, catalysts, and activation methods, ˙OH generation can be broadly categorized into three recurring mechanistic routes that occur across different subsets of AOP systems: oxidant activation, ˙OH generation from water through oxidation or dissociation, and transition-metal redox cycling.

Over the past decades, significant advances in catalyst engineering and reactor design have enhanced ˙OH production through approaches such as defect engineering, heteroatom doping, oxygen-vacancy construction, single-atom catalysis, and heterojunction design.^6,28–34^ Meanwhile, increasing attention has been devoted to ROS networks involving ˙OH, SO_4_˙^−^, O_2_˙^−^, ^1^O_2_, reactive chlorine species, and high-valent metal intermediates.^16–20,35,36^ However, current research remains largely fragmented because AOPs are typically classified according to oxidants or technologies rather than the fundamental mechanisms governing ˙OH generation and utilization. Furthermore, catalyst performance is often evaluated based on pollutant removal or radical production, while the efficiency with which generated ˙OH participates in productive oxidation receives comparatively little attention. Emerging oxidants such as peroxymonocarbonate (HCO_4_^−^), formed *in situ* from HCO_3_^−^ and H_2_O_2_, have recently attracted attention as alternative precursors for ROS generation. Selective catalytic or photocatalytic activation of HCO_4_^−^ can promote O–O bond cleavage and generate ˙OH and CO_3_˙^−^, while catalyst composition and activation pathways can regulate the resulting ROS distribution.^37–40^

Although numerous reviews have summarized individual AOP technologies, a unified framework linking ˙OH formation, ROS interactions, catalyst design, and practical performance is still lacking.^41–44^ Therefore, this review proposes a ˙OH-centered perspective that integrates diverse AOPs through three formation pathways: oxidant activation, ˙OH generation from water through oxidation or dissociation, and metal redox cycling. Particular emphasis is placed on ˙OH utilization efficiency, ROS interactions, water-matrix effects, and catalyst-design principles. By shifting the focus from oxidant-specific classifications to rational ˙OH engineering, this review provides a conceptual framework for developing more efficient and scalable next-generation AOPs.

## 2. Kinetic behavior and reactivity principles of hydroxyl radicals toward organic compounds

Hydroxyl radicals are the most reactive oxidizing species encountered in aqueous AOPs. Their exceptionally high redox potential (1.8–2.7 V) and high reactivity enable reactions with many aqueous compounds to approach the diffusion-controlled limit (Table S1).^45–47^ Consequently, second-order rate constants (*k*_2_) for reactions between ˙OH and organic contaminants commonly fall within the range of 10^8^–10^10^ M^−1^ s^−1^ (Table S2), explaining the broad applicability of ˙OH-based AOPs for degrading pharmaceuticals, pesticides, dyes, perfluorinated compounds, and natural organic matter.^45^

Although ˙OH is often described as a non-selective oxidant, its reactivity is not entirely random. The efficiency of pollutant degradation depends strongly on molecular electronic structure, reaction pathways, and environmental conditions. Understanding these kinetic and mechanistic principles is therefore essential for interpreting ˙OH utilization in different AOPs and for developing rational catalyst-design strategies discussed in later sections.

### 2.1. Fundamental reaction pathways of ˙OH

**Fig. 1:**
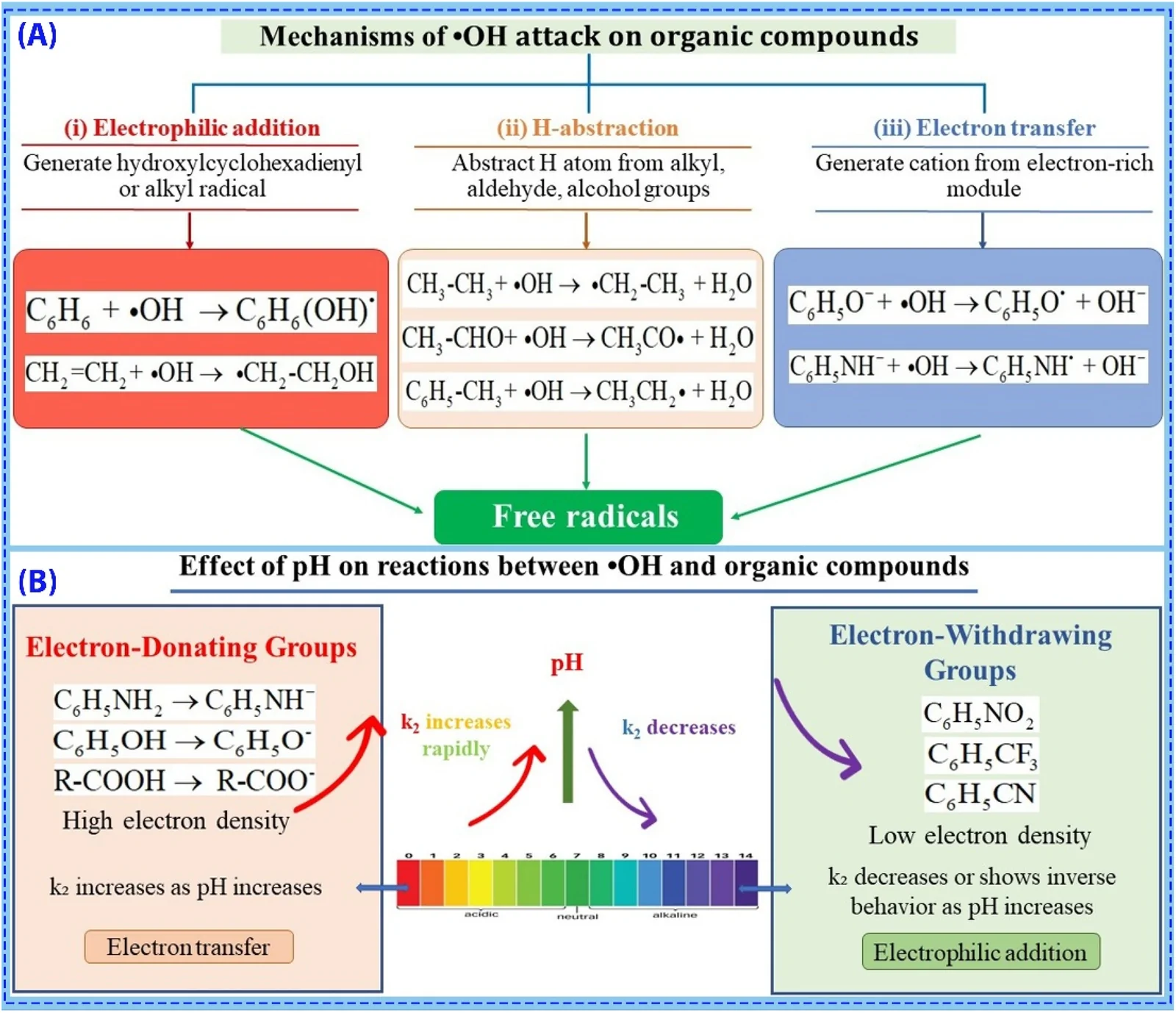
(A) Reaction pathways of ˙OH attack. (B) Effect of pH on *k*_2_ for ˙OH reactions with organic compounds.

The oxidation of organic compounds by ˙OH generally proceeds through three principal pathways (Fig. 1A).^41,45–47^ Recent studies in catalytic ozonation, photocatalysis, and electrochemical oxidation have further confirmed that these pathways dominate the degradation of a wide range of emerging contaminants, pharmaceuticals, and pesticides.^48,49^

#### 2.1.1 Electrophilic addition

Because ˙OH possesses strong electrophilic character, it preferentially attacks electron-rich aromatic rings and unsaturated C=C bonds, producing hydroxylated radical intermediates. These intermediates subsequently undergo oxygenation, rearrangement, and ring-opening reactions, ultimately leading to fragmentation and mineralization.^45,50,51^

#### 2.1.2 Hydrogen abstraction

For aliphatic compounds and molecules containing activated C–H bonds, ˙OH can abstract hydrogen atoms to generate carbon-centered radicals. These radicals readily react with dissolved oxygen, initiating chain oxidation reactions that progressively convert organic compounds into smaller oxygenated products.^46,52,53^

#### 2.1.3 Electron transfer

Electron-rich organic molecules may undergo direct electron transfer to ˙OH, forming transient radical cations that rapidly decompose or participate in subsequent oxidation reactions.^41,47^ The coexistence of these pathways explains the extraordinary oxidation capability of ˙OH and its effectiveness toward structurally diverse contaminants. More importantly, the dominant pathway often determines the subsequent degradation route, product distribution, and mineralization efficiency.^54^

### 2.2. Experimental and computational determination of ˙OH reactivity

Because of the high reactivity and hence ultrashort lifetime of ˙OH, direct kinetic characterization remains challenging. Several complementary approaches have therefore been developed to determine ˙OH reaction kinetics.

**Fig. 2:**
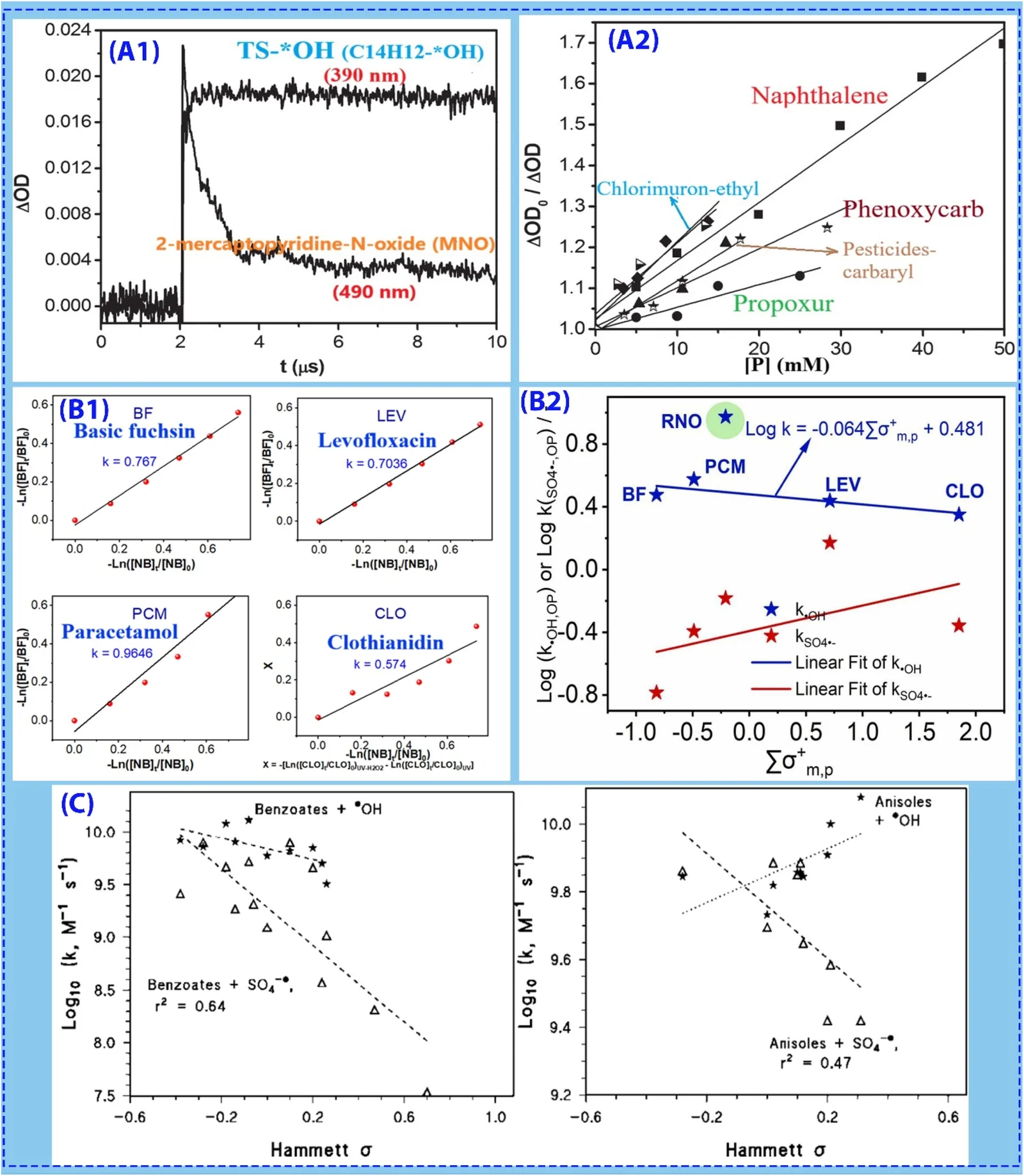
(A1) Optical density of TS-˙OH and MNO at 390 and 490 nm, respectively, and (A2) Stern–Volmer plots for target organics relative to TS.^56^ (B1) Kinetic competition between pollutants and NB.^61^ Correlations between ln(*k*_2_) of ˙OH or SO_4_˙^−^ and ∑*σ*_m,p_^+^ are shown in (B2)^61^ and (C),^68^ respectively. Panels A1 and A2 were adapted with permission from ref. 56. Panels B1 and B2 were reproduced with permission from ref. 61. Panel C was reproduced with permission from ref. 68. Copyright © Wiley, Elsevier, and American Chemical Society, respectively.

Pulse radiolysis and flash photolysis are important complementary techniques for investigating transient radical species and determining reaction kinetics on nanosecond-to-microsecond timescales. These approaches can provide reliable second-order rate constants when appropriate kinetic models and experimental conditions are applied.^45,55^ In particular, *trans*-stilbene (TS) is frequently used as a reference probe, where competitive scavenging between TS and target compounds allows determination of *k*_2_ values through transient absorption measurements (Fig. 2A1&A2).^56^

For practical AOP systems, competition kinetics methods are more commonly applied. In these approaches, the reactivity of target contaminants is quantified relative to well-established reference compounds such as nitrobenzene (NB) (Fig. 2B1).^57–59^

In parallel, quantitative structure–activity/property relationship (QSAR/QSPR) models have emerged as valuable predictive tools (Fig. 2B2). These approaches correlate experimentally measured *k*_2_ values with molecular descriptors such as frontier orbital energies, atomic charges, steric parameters, and electronic substituent effects, enabling estimation of ˙OH reactivity for contaminants lacking experimental data.^60,61^ Collectively, these experimental and computational methods provide the quantitative foundation for linking molecular structure with ˙OH oxidation behavior.

### 2.3. Structure–reactivity relationships governing ˙OH oxidation

Recent studies demonstrate that ˙OH reactivity is governed primarily by molecular electronic structure rather than simply by contaminant class. For aromatic compounds, significant linear relationships have been established between ln(*k*_2_) and Hammett substituent constants (Σ*σ*_m,p_^+^) (Fig. 2B2&C).^60,61^ Electron-donating substituents increase electron density on aromatic rings and facilitate electrophilic ˙OH attack, whereas electron-withdrawing substituents reduce ring electron density and suppress reactivity.^45,62,63^ Consequently, phenols, anilines, and phenoxy-containing pharmaceuticals often exhibit reaction rate constants approaching 10^10^ M^−1^ s^−1^, while nitro-, cyano-, and trifluoromethyl-substituted aromatic compounds react substantially more slowly.^64–67^ These observations establish molecular structure–reactivity relationships as a mechanistic framework for predicting contaminant degradation kinetics and identifying the molecular features that control ˙OH attack.

### 2.4. Influence of solution chemistry on ˙OH reactivity

Beyond molecular structure, solution chemistry strongly influences ˙OH oxidation kinetics. Among environmental variables, pH plays a particularly important role because it alters contaminant speciation and electronic structure (Fig. 1B). Deprotonation of phenolic, carboxylic, or amine-containing compounds generally increases electron density, facilitating electrophilic addition and electron-transfer reactions and leading to higher *k*_2_ values under alkaline conditions.^45,62,63^ Conversely, for compounds containing strongly electron-withdrawing substituents, ionization may reduce aromatic electron density and decrease reactivity.^64,65^ Therefore, pH effects on ˙OH oxidation arise from the combined influence of protonation state, molecular electronic structure, and reaction-pathway selection.

## 3. Unified mechanistic framework for hydroxyl radical generation across AOPs

### 3.1. Recurring mechanistic pathway I: ˙OH generation *via* oxidant bond cleavage

**Fig. 3:**
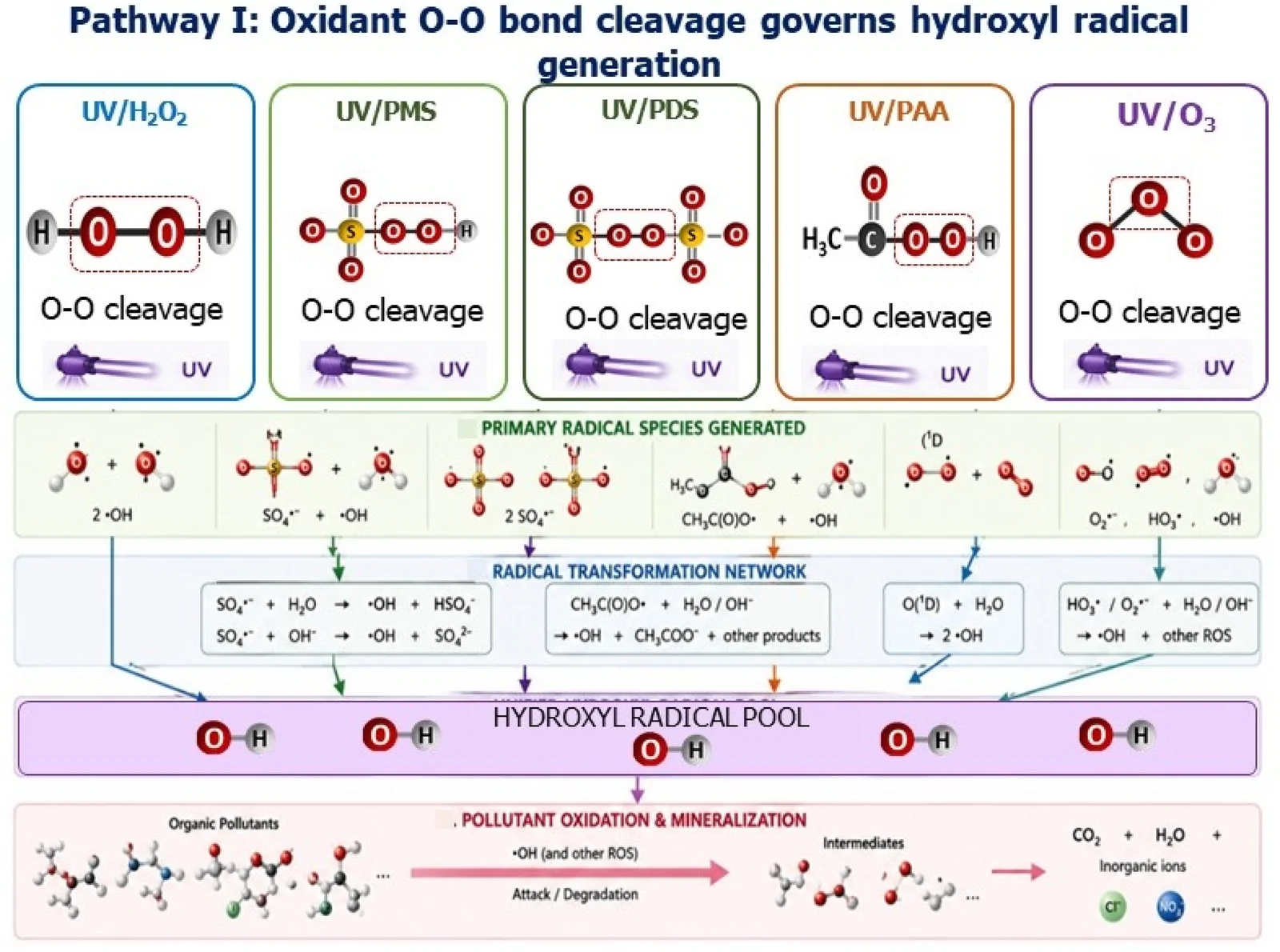
Unified mechanistic framework for ˙OH generation across representative UV-AOPs.

Hydroxyl radicals are the most widely recognized reactive species responsible for contaminant oxidation in AOPs. Despite the diversity of oxidants and activation technologies employed in UV-AOPs, many systems ultimately rely on a common mechanistic framework in which oxidant activation initiates the formation of reactive intermediates that subsequently generate ˙OH (Fig. 3).^3,4,20,69^

Representative oxidants including hydrogen peroxide (H_2_O_2_), peroxymonosulfate (PMS), peroxydisulfate (PDS), peracetic acid (PAA), ozone (O_3_) and the emerging oxidant peroxymonocarbonate (HCO_4_^−^) differ substantially in molecular structure and activation requirements.^16,20,70–72^ Depending on the oxidant chemistry, activation may generate ˙OH directly or produce intermediate ROS that subsequently participate in reactions leading to ˙OH formation (Table 1).^73,74^

**Table 1:** O–O bond cleavage pathways in representative UV/AOPs

System	Initial activation step	Primary radicals	Route to ˙OH
UV/H_2_O_2_	H_2_O_2_ + *hν* → 2˙OH	˙OH	Direct
UV/PMS	HSO_5_^−^ + *hν* → SO_4_˙^−^ + ˙OH	SO_4_˙^−^, ˙OH	Direct + secondary
UV/PDS	S_2_O_8_^2−^ + *hν* → 2SO_4_˙^−^	SO_4_˙^−^	SO_4_˙^−^ + H_2_O/OH^−^ → ˙OH
UV/PAA	CH_3_C(O)OOH + *hν* → CH_3_C(O)O˙ + ˙OH	CH_3_C(O)O˙, ˙OH	Direct
O_3_/UV	O_3_ + *hν* → O(^1^D) + O_2_	O(^1^D)	O(^1^D) + H_2_O → 2˙OH
O_3_/H_2_O_2_	Peroxone chain reactions	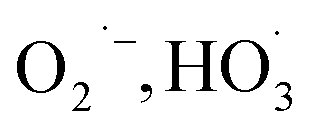	Secondary ˙OH

From a ˙OH-centered perspective, the efficiency of AOPs is therefore closely linked to the ability of a system to convert stored oxidizing power into a reactive ˙OH pool available for pollutant degradation.^75^ This recurring mechanistic pathway involves two fundamental mechanistic stages: (i) activation of oxidant-bound oxygen species through bond cleavage and electron-transfer processes, and (ii) transformation and interconversion of primary ROS into a common ˙OH-centered oxidation network.

#### 3.1.1 Molecular mechanisms of O–O bond activation

The unified framework proposed above suggests that many oxidant-based AOPs share a common molecular origin for ˙OH generation: activation and cleavage of peroxide O–O bonds.^16,20,70,76,77^ For peroxide-based oxidants, this initial step converts stored oxidative potential into reactive intermediates that directly or indirectly lead to ˙OH formation (Fig. 3). In peroxide-based systems, O–O bond cleavage can be induced through photochemical, catalytic, thermal, or electrochemical activation. Under UV irradiation, peroxide oxidants undergo homolytic bond scission (Table 1), representing the primary initiation step in UV/H_2_O_2_, UV/PMS, UV/PDS, and UV/PAA systems.^3,35,59,69,78,79^

At the molecular level, O–O bond activation is commonly driven by electron transfer into the antibonding σ* orbital of peroxide species. This process weakens the peroxide linkage, elongates the O–O bond, and lowers the energy barrier for bond dissociation, thereby facilitating subsequent ˙OH generation.^16,20,70^ The efficiency and selectivity of O–O bond activation depend strongly on interfacial charge transfer and active-site structure. In emerging peroxymonocarbonate (HCO_4_^−^) systems, selective O–O bond activation promotes ˙OH and CO_3_˙^−^ generation while suppressing competing H_2_O_2_ decomposition.^72,80^ Recent DFT studies further show that selective HCO_4_^−^ adsorption and interfacial electron transfer at Co/NSC sites can elongate the O–O bond and facilitate its cleavage, providing molecular-level evidence for preferential HCO_4_^−^ activation over H_2_O_2_ decomposition.^38^ Consequently, catalyst surfaces capable of promoting interfacial charge transfer can substantially accelerate oxidant activation and increase ˙OH production. Such mechanisms are widely observed in transition-metal catalysts, defect-engineered materials, and single-atom catalytic systems.^16,17,20,74,81,82^

**Fig. 4:**
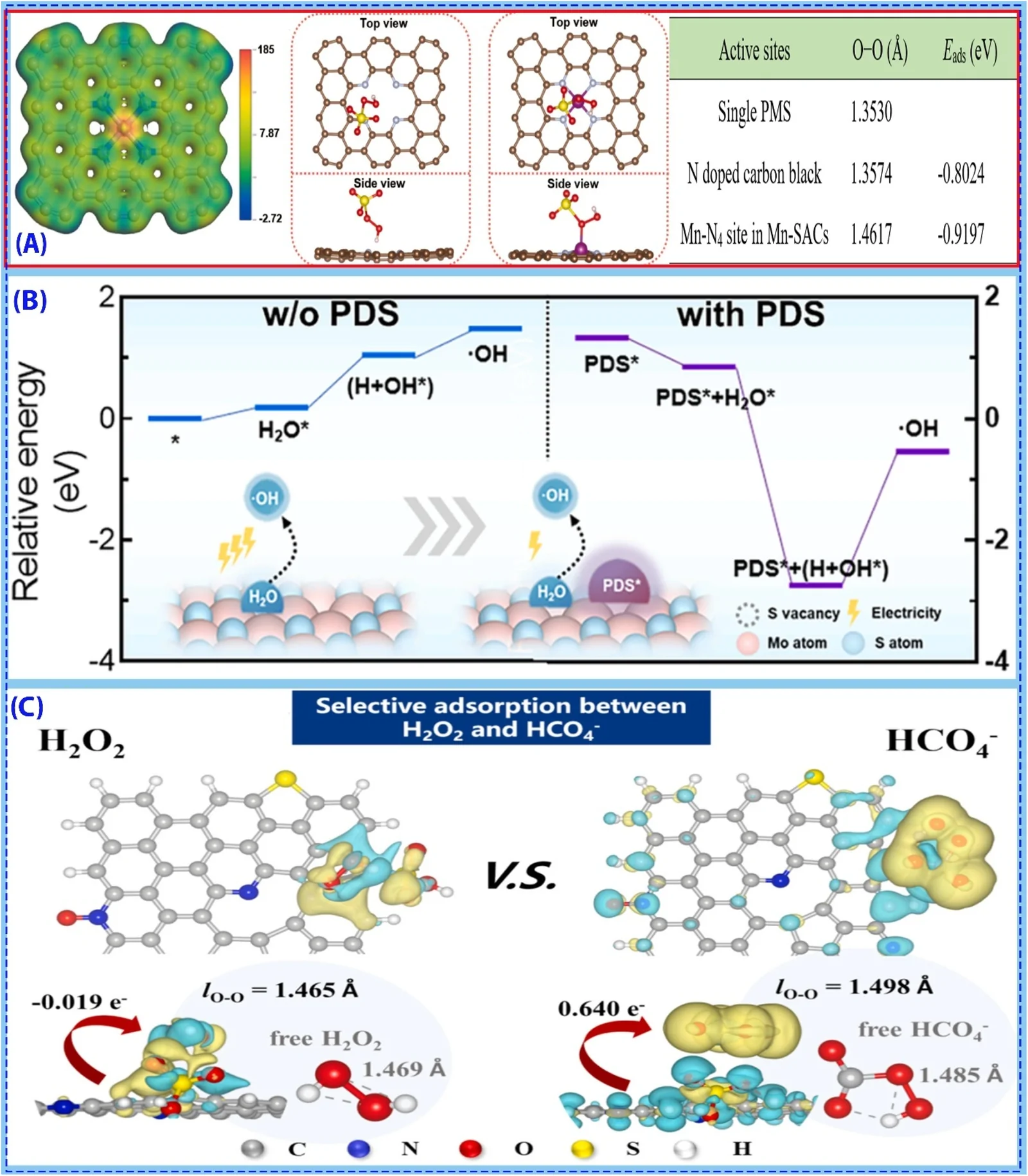
(A) Charge distribution of Mn–N_4_ and PMS adsorption configurations on N-carbon black and Mn–N_4_.^17^ (B) Free-energy changes for ˙OH formation in the presence of PDS.^83^ (C) Calculated charge-density variations and adsorption energies of H_2_O_2_ and HCO_4_^−^ interacting with –SO_3_H functional sites on NSC.^38^ Panels A, B, and C were reproduced with permission from ref. 17 and 83, and,^38^ respectively. Copyright © Elsevier.

As illustrated in Fig. 4A, electron donation from redox-active metal centers to adsorbed PMS promotes O–O bond elongation and peroxide activation.^16,17,20,75^ DFT calculations further reveal that electronic-structure engineering can reduce the thermodynamic barrier for ˙OH formation. For example, sulfur vacancies in molybdenite enhance oxidant adsorption and charge transfer, while adsorbed PDS* significantly lowers the Gibbs free energy required for ˙OH generation (Fig. 4B).^83^

Collectively, these findings demonstrate that the efficiency of ˙OH production is strongly influenced by the ability of catalysts to facilitate electron transfer and O–O bond dissociation. Therefore, engineering catalyst electronic structure to promote oxidant activation represents a broadly applicable strategy for enhancing ˙OH generation across diverse peroxide-based AOPs.^16,17,20,81,84^

#### 3.1.2 Radical convergence toward a unified hydroxyl radical pool

**Fig. 5:**
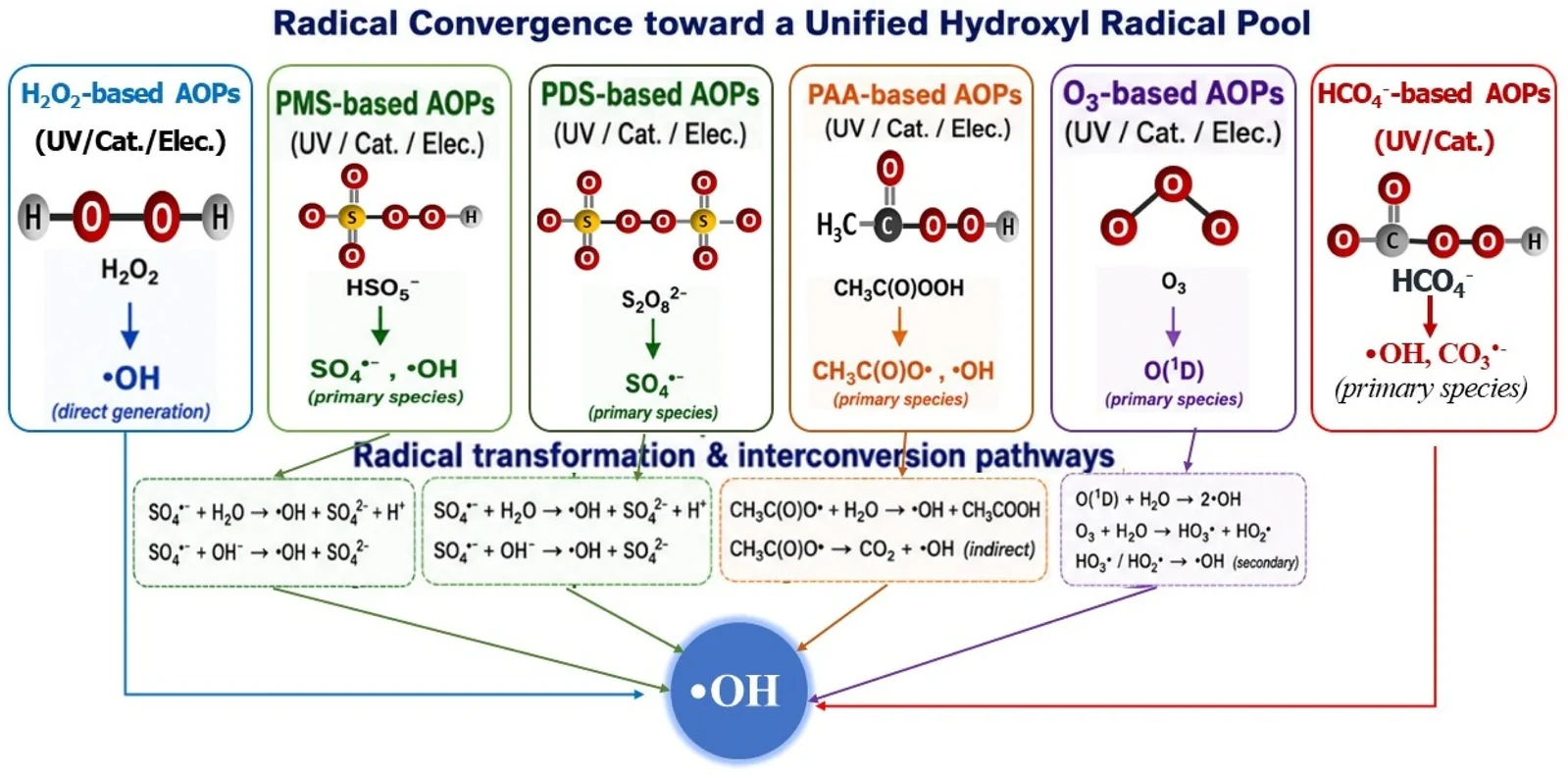
Radical convergence toward a unified ˙OH pool across AOPs.

Although different oxidants generate distinct primary reactive species during activation, these species rarely remain independent throughout the oxidation process. HCO_4_^−^-based systems further illustrate this ROS-network behavior. Selective HCO_4_^−^ activation can generate ˙OH together with CO_3_˙^−^, introducing carbonate-radical chemistry into the ˙OH-centered oxidation network. The relative contributions of these species depend on oxidant composition, catalyst structure, pH, and activation conditions, demonstrating that oxidant activation selectivity can determine not only the amount of ˙OH generated but also the composition of the surrounding ROS network. Instead, they undergo rapid transformation, propagation, and interconversion reactions that progressively enrich the ˙OH population, creating a common ˙OH-centered oxidation environment (Fig. 5).^16,20,21,69,73,85,86^

Hydrogen peroxide-based AOPs represent the most direct pathway, as ˙OH is generated immediately during oxidant activation and dominates oxidation from the outset.^70,78,79,87,88^ In contrast, activation of PMS and PDS initially produces SO_4_˙^−^, which subsequently react with water, hydroxide ions, and other reactive species to generate ˙OH through secondary conversion pathways (eqn (1) and (2)).^16,20,69^ Consequently, although SO_4_˙^−^ radicals may dominate the initiation stage, a substantial fraction of oxidative activity is eventually transferred to ˙OH.^82^

Related ROS transformation processes occur in ozone-based AOPs. UV photolysis of ozone generates electronically excited oxygen atoms, O(^1^D), which rapidly react with water to produce ˙OH (eqn (3)–(7)).^21,48,54,89–91^ Likewise, oxidants such as PAA may initially form organic radicals, but subsequent reaction cascades can also contribute to ˙OH generation under appropriate conditions (Table 1).^35,92^ Together with the HCO_4_^−^ pathway described above (eqn (8)), these examples illustrate that oxidant-specific activation can generate distinct primary ROS while subsequently feeding into interconnected oxidation networks.^39,93^

The extent of convergence depends strongly on reaction conditions. For example, alkaline environments promote the conversion of SO_4_˙^−^ to ˙OH, whereas acidic conditions favor sulfate-radical persistence.^3,94,95^ Therefore, the relative abundance of ˙OH is controlled not only by oxidant activation but also by subsequent ROS transformation reactions.^96,97^

Taken together, H_2_O_2_, PMS, PDS, PAA, ozone, and HCO_4_^−^ represent different sources or precursors of oxidative potential that generate distinct primary ROS and, under appropriate conditions, contribute to a broader ˙OH-centered oxidation network. This radical convergence framework provides a ˙OH-centered perspective for interpreting AOPs, emphasizing the evolution of reaction networks toward ˙OH generation and utilization during contaminant degradation.

1 S_2_O_8_^2−^ + *hν* → 2SO_4_˙^−^

2 SO_4_˙^−^ + H_2_O/OH^−^ → ˙OH + HSO_4_^−^/SO_4_^2−^

3 O_3_ + *hν* → O_2_ + O(^1^D)

4 O(^1^D) + H_2_O → 2˙OH

5
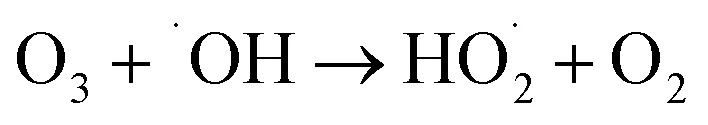

6 O_3_ + O_2_˙^−^ → O_3_˙^−^ + O_2_

7
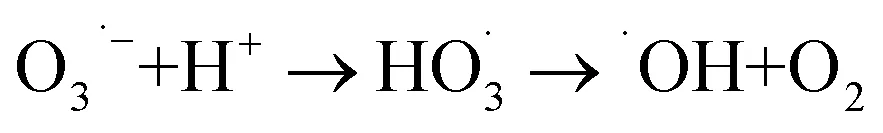
8
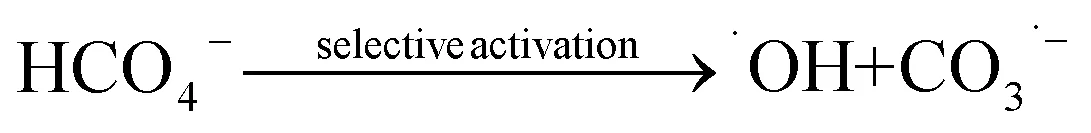


### 3.2. Recurring mechanistic pathway II: ˙OH generation from water

**Fig. 6:**
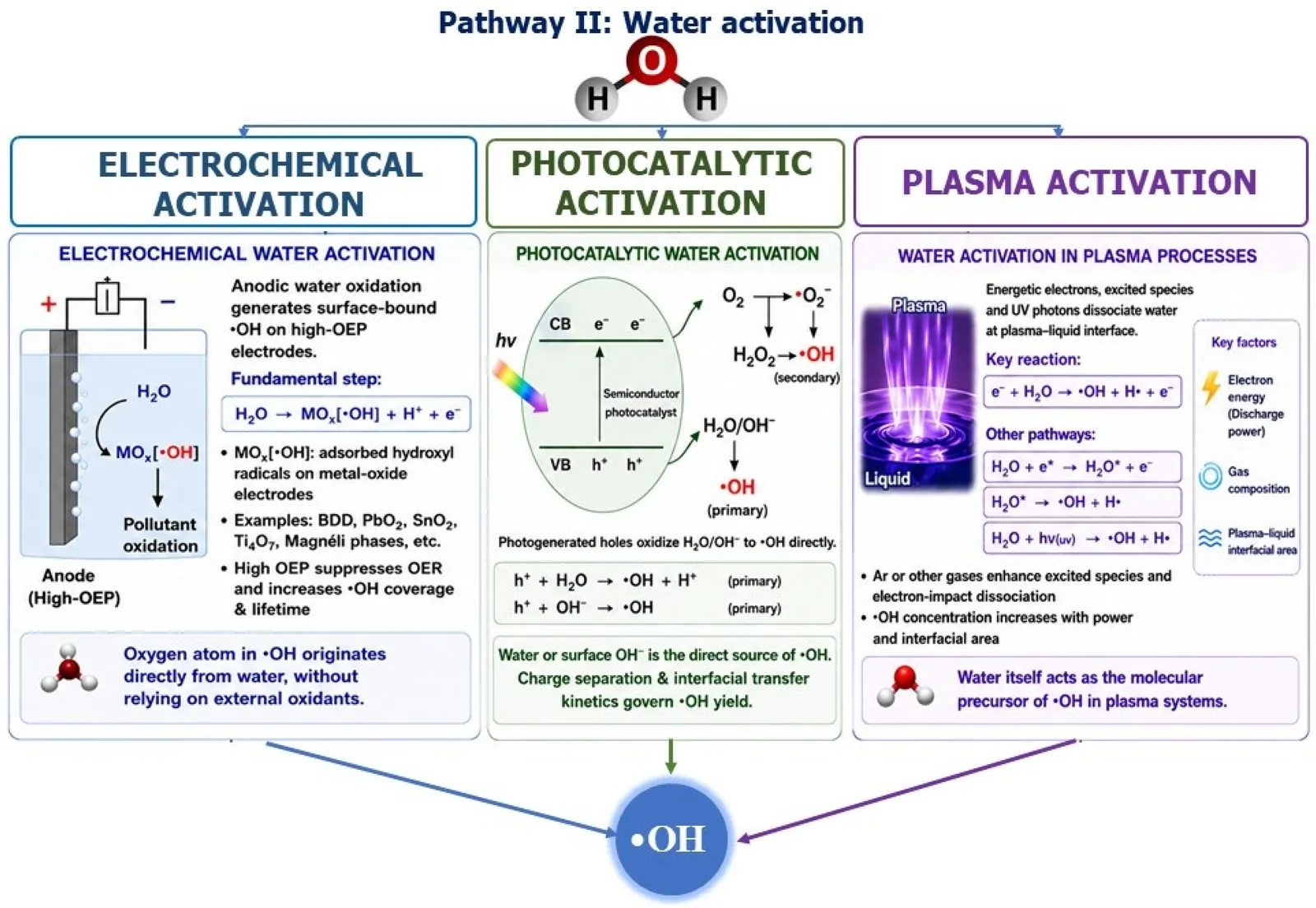
Representative pathways for ˙OH generation from water in AOPs.

In contrast to oxidant-activation pathways, hydroxyl radicals in several advanced oxidation technologies originate directly from water molecules or hydroxide ions. In these systems, water serves not merely as the reaction medium but as the primary precursor of ˙OH. Rather than relying on peroxide bond cleavage, radical generation can proceed through electrochemical or photocatalytic oxidation of water, or through plasma-induced water dissociation. Consequently, electrochemical oxidation, photocatalysis, and plasma processes can be unified within a second mechanistic pathway in which water itself becomes the source of hydroxyl radicals.^98,99^Fig. 6 summarizes this unified framework for ˙OH generation from water, highlighting how electrochemical, photocatalytic, and plasma systems employ different activation mechanisms but ultimately produce a common ˙OH-centered oxidation environment.

#### 3.2.1 Electrochemical water oxidation and ˙OH generation

Electrochemical oxidation provides one of the clearest examples of ˙OH generation directly from water molecules rather than from external oxidants (Fig. 6). Unlike peroxide-based AOPs, where radical formation often involves O–O bond cleavage, electrochemical systems can produce ˙OH through anodic one-electron oxidation of water (or water discharge) without requiring H_2_O_2_, PMS, PDS, or ozone as radical precursors.^11–15,100^

The fundamental step involves the anodic oxidation of water at the electrode surface (Table 2). According to the Comninellis model, water oxidation initially generates surface-bound hydroxyl radicals adsorbed on metal-oxide electrodes:^11,15^

9 M + H_2_O → M(˙OH)_ads_ + H + e^−^

where M(˙OH)_ads_ represents a surface-associated hydroxyl species formed on the electrode surface. These highly reactive intermediates subsequently participate in pollutant oxidation through direct reactions at or near the electrode surface.^11–15^

**Table 2:** Representative pathways of ˙OH generation from water in AOPs

Technology	Initial activation step	Immediate precursor	Route to ˙OH
Electrochemical oxidation	H_2_O → ˙OH + H^+^ + e^−^	H_2_O	Direct
Photocatalysis	h^+^ + H_2_O/OH^−^ → ˙OH	H_2_O/OH^−^	Direct
Plasma	e^−^ + H_2_O → ˙OH + H˙ + e^−^	H_2_O	Direct
Photoelectrocatalysis	h^+^ + H_2_O/OH^−^ → ˙OH	H_2_O/OH^−^	Direct

This water-derived ˙OH pathway has been reported for a range of high-oxygen-evolution-potential (OEP) anodes, including boron-doped diamond (BDD),^101^ PbO_2_,^57,102^ SnO_2_,^103^ Ti_4_O_7_ and other Magnéli-phase electrodes.^11–14^ Although these materials differ substantially in crystal structure, conductivity, and surface chemistry, their electrochemical oxidation behavior can involve ˙OH formation through anodic activation or discharge of water.^73,104^ High-OEP electrodes can favor ˙OH-mediated oxidation by reducing the relative contribution of the competing OER under appropriate operating conditions, thereby improving the availability of hydroxyl intermediates for pollutant oxidation.^12–15^

Recent studies further demonstrate that electrode composition, defect structures, and electronic properties can influence the formation, adsorption, and reactivity of surface hydroxyl species. Defect-rich metal oxides, doped electrodes, and conductive Magnéli phases can facilitate charge transfer and optimize the balance between ˙OH generation and oxygen evolution, thereby affecting oxidation performance.^101,103,105^ In electrochemical oxidation, the oxygen atom incorporated into the initially generated ˙OH originates from water, establishing water as the molecular precursor of the hydroxyl radical. This distinguishes the water-derived ˙OH pathway from AOPs in which ˙OH is generated primarily through activation or transformation of externally supplied oxidants.^11–15,100^

#### 3.2.2 Photocatalytic water oxidation and ˙OH generation

Photocatalytic systems establish a fundamentally distinct pathway for ˙OH generation compared with peroxide-based advanced oxidation processes. Photocatalysis enables direct formation of ˙OH from water molecules or surface hydroxyl species through photogenerated charge carriers.^5–10,106–108^ Consequently, the oxygen atom in ˙OH is derived directly from H_2_O or OH^−^ adsorbed on the catalyst surface rather than from externally added oxidants (Fig. 6).

Upon irradiation with photons possessing energies equal to or greater than the semiconductor band gap, photocatalysts such as g-C_3_N_4_,^6^ ZnO,^5^ Bi_2_WO_6_@Ti_2_C,^108^ Fe_3_O_4_@MnFe,^28^ g-C_3_N_4_@NMOF,^109^ and PANI@g-C_3_N_5_/Gd_2_O_3_ (PANI: polyaniline)^9^ generate conduction-band electrons (e_CB_^−^) and valence-band holes (h_VB_^+^) (eqn (10)). Owing to their highly positive valence-band potentials, photogenerated holes can directly oxidize adsorbed H_2_O or OH^−^ to produce ˙OH radicals (eqn (11) and (12)). This hole-mediated oxidation of H_2_O or surface hydroxyl species represents an important pathway for ˙OH formation in photocatalytic systems.^6,7,10,87^

In addition, photogenerated electrons may reduce dissolved oxygen to superoxide radicals (˙O_2_^−^) (eqn (13)), which subsequently undergo proton-coupled electron-transfer reactions to form H_2_O_2_ and ultimately additional ˙OH (eqn (14)).^106,110^ Although this oxygen-reduction pathway contributes to overall radical production, direct oxidation of H_2_O or surface hydroxyl species by photogenerated holes represents an important mechanistic route for water-derived ˙OH generation in photocatalytic AOPs.

10 Photocatalyst + *hν* → e^−^ + h^+^

11 h^+^ + H_2_O → ˙OH + H^+^

12 h^+^ + OH^−^ → ˙OH

13 O_2_ + e^−^ → O_2_˙^−^

14 O_2_˙^−^ + H_2_O → ˙OH + OH^−^

The efficiency of ˙OH generation in photocatalytic systems is strongly governed by charge-carrier separation and interfacial transfer kinetics. Strategies such as heterojunction construction, Z-scheme architectures, oxygen-vacancy engineering, defect modulation, and single-atom active-site incorporation effectively suppress electron–hole recombination and enhance surface-hole availability for interfacial oxidation of H_2_O/OH^−^ and ˙OH generation. ^5,7–10,106–108^ Representative examples include oxygen-vacancy-rich Bi-based photocatalysts, g-C_3_N_4_-derived heterostructures, Ti-based photo-Fenton catalysts, and Z-scheme semiconductor systems, which can enhance ˙OH production by facilitating ˙OH generation from water.^106–108,111,112^

Therefore, photocatalytic systems demonstrate that water itself can serve as the direct molecular precursor of ˙OH, with photogenerated holes promoting the oxidation of H_2_O/OH^−^ and thereby contributing to ˙OH generation as a recurring mechanistic pathway, alongside electrochemical water-derived ˙OH generation (Fig. 6).

#### 3.2.3 Plasma-induced water dissociation and ˙OH generation

Plasma-based advanced oxidation processes (plasma-AOPs) provide one of the most direct demonstrations of ˙OH generation from water (Fig. 6). Unlike H_2_O_2_-, PMS-, PDS-, PAA-, or ozone-based systems, plasma does not necessarily require external oxidants to produce ˙OH. Instead, hydroxyl radicals are generated directly through the dissociation of water molecules by energetic electrons, excited species, ultraviolet photons, and strong electric fields generated within the plasma phase.^113–116^

The primary mechanism involves electron-impact dissociation of water:

15 H_2_O + e^−^ → ˙OH + ˙H

High-energy electrons collide with H_2_O molecules, producing ˙OH and hydrogen atoms. Simultaneously, water molecules can be excited to electronically excited states (H_2_O*) (eqn (16)–(19)), which subsequently undergo dissociation to yield additional ˙OH.^113,114^ Plasma-generated UV photons and excited species can also induce direct photolytic dissociation of water, further contributing to ˙OH formation.^117^ Consequently, water itself acts as the molecular precursor of ˙OH, without the need for O–O bond cleavage or oxidant activation.

**Fig. 7:**
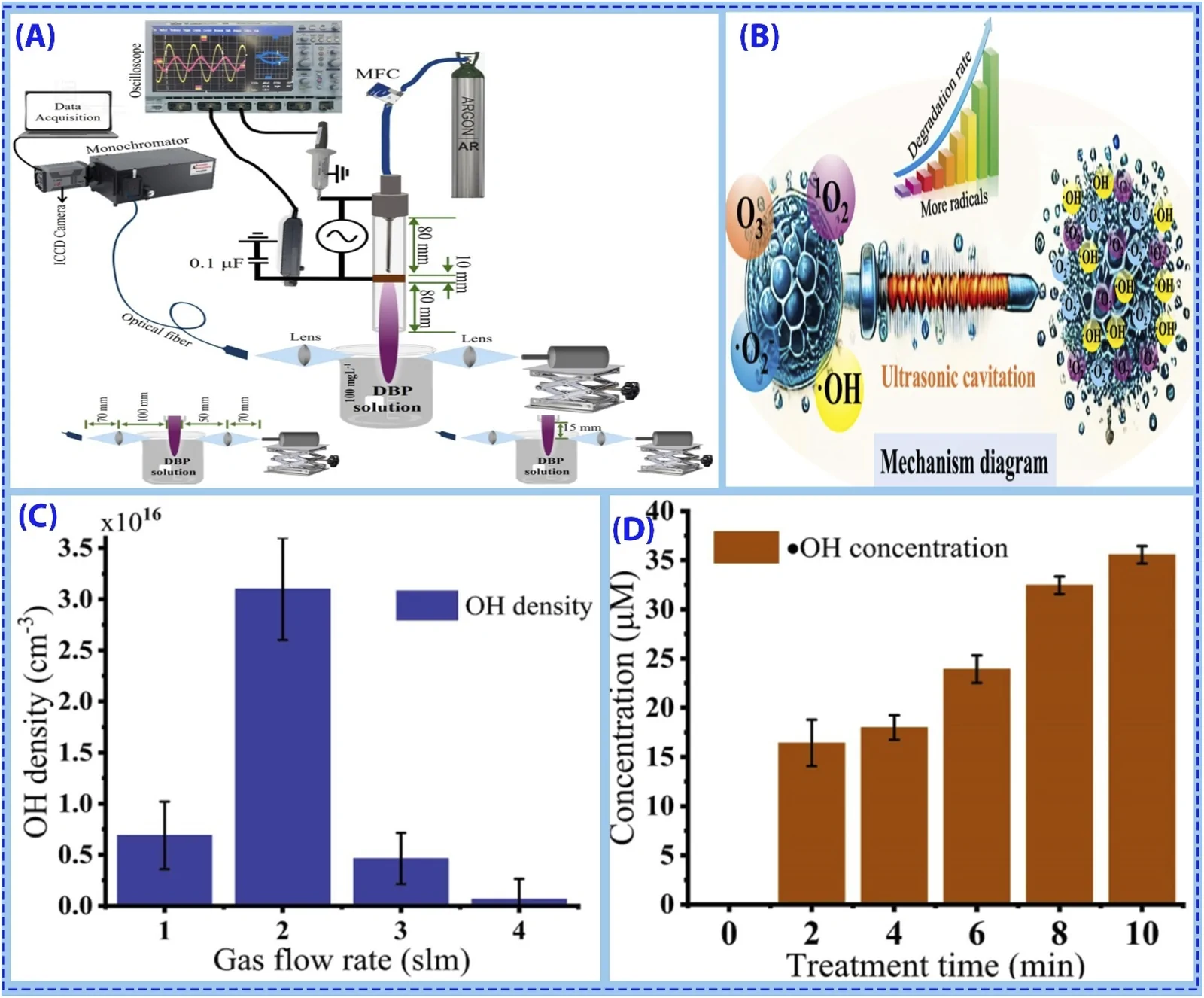
(A) Plasma catalytic system.^117^ (B) Radical generation mechanisms in plasma-ultrasonic systems.^113^ (C) Maximum ˙OH density at a flow rate of 2 slm gas.^117^ (D) Time-dependent ˙OH concentration.^117^ Panels A, C, and D were reproduced with permission from ref. 117, and panel B was reproduced with permission from ref. 113. Copyright © Elsevier.

Atmospheric-pressure plasma systems operated with Ar or other carrier gases further enhance water dissociation through the generation of excited species and energetic electrons (eqn (20)–(23)).^117^ As a result, plasma-liquid interfaces become effective reaction zones for continuous ˙OH production.^118^ Representative plasma-induced radical-generation pathways are illustrated in Fig. 7A&B.

16 H_2_O + e^−^ → H_2_O^+^ + 2e^−^

17 H_2_O^+^ → H^+^ + ˙OH

18 H_2_O + e^−^ → H_2_O*

19 H_2_O* → ˙H + ˙OH

20 e^−^ + Ar → Ar* + e^−^

21 Ar* + H_2_O → Ar + ˙OH + H^+^

22
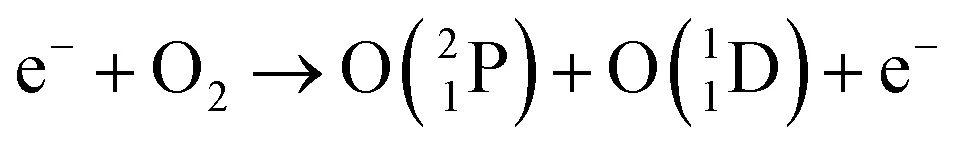
23
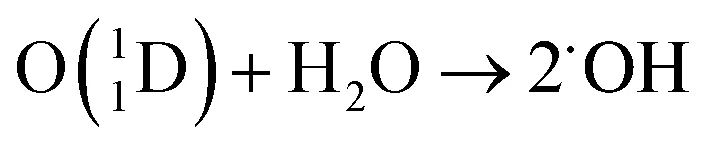


The efficiency of plasma-induced ˙OH generation depends primarily on electron energy, discharge power, gas composition, and plasma-liquid interfacial area. Increasing applied voltage or optimizing gas flow rate enhances electron-impact reactions and increases ˙OH production, whereas excessive operating intensity may reduce energy efficiency due to competing recombination processes (Fig. 7C).^117,119^ Plasma-generated ˙OH concentrations typically increase continuously during treatment, reaching 35.5 µM after 10 min of operation (Fig. 7D), confirming sustained water dissociation at the plasma-liquid interface.^117^

### 3.3. Recurring mechanistic pathway III: metal redox cycling

#### 3.3.1 Fundamental mechanism of metal redox cycling

Unlike oxidant activation and water-derived ˙OH generation pathways, metal-mediated AOPs generate ˙OH through continuous electron transfer between different oxidation states of transition metals. In these systems, transition metals do not primarily act as oxidants themselves; instead, they function as catalytic electron shuttles that repeatedly activate oxidants and sustain ˙OH production throughout the reaction process.^16–20^

The fundamental mechanism involves reversible redox transitions between low- and high-valence metal species:

24 M^*n*+^ + e^−^ ↔ M^(*n*−1)+^ (M: Fe, Co, Mn, Cu)

Through these redox cycles, reduced metal species continuously transfer electrons to oxidants such as H_2_O_2_, PMS, PDS, and dissolved oxygen, promoting bond cleavage and generating radical intermediates that directly or indirectly yield ˙OH.^16,17,20,78^ Simultaneously, the oxidized metal species are regenerated through subsequent electron-transfer reactions, establishing a self-sustaining catalytic cycle.

A representative example is the Fenton reaction, where Fe^2+^ activates H_2_O_2_ to produce ˙OH:

25 Fe^2+^ + H_2_O_2_ → Fe^3+^ + ˙OH + OH^−^

Similar mechanisms operate in Co-, Cu-, and Mn-based catalytic systems, where metal redox cycling continuously drives oxidant activation and maintains ˙OH generation.^17,78,97,120^ Therefore, the effectiveness of metal-mediated AOPs is largely determined by the efficiency of electron transfer and the regeneration rate of active redox centers rather than by the intrinsic oxidizing power of the metal species themselves.

**Fig. 8:**
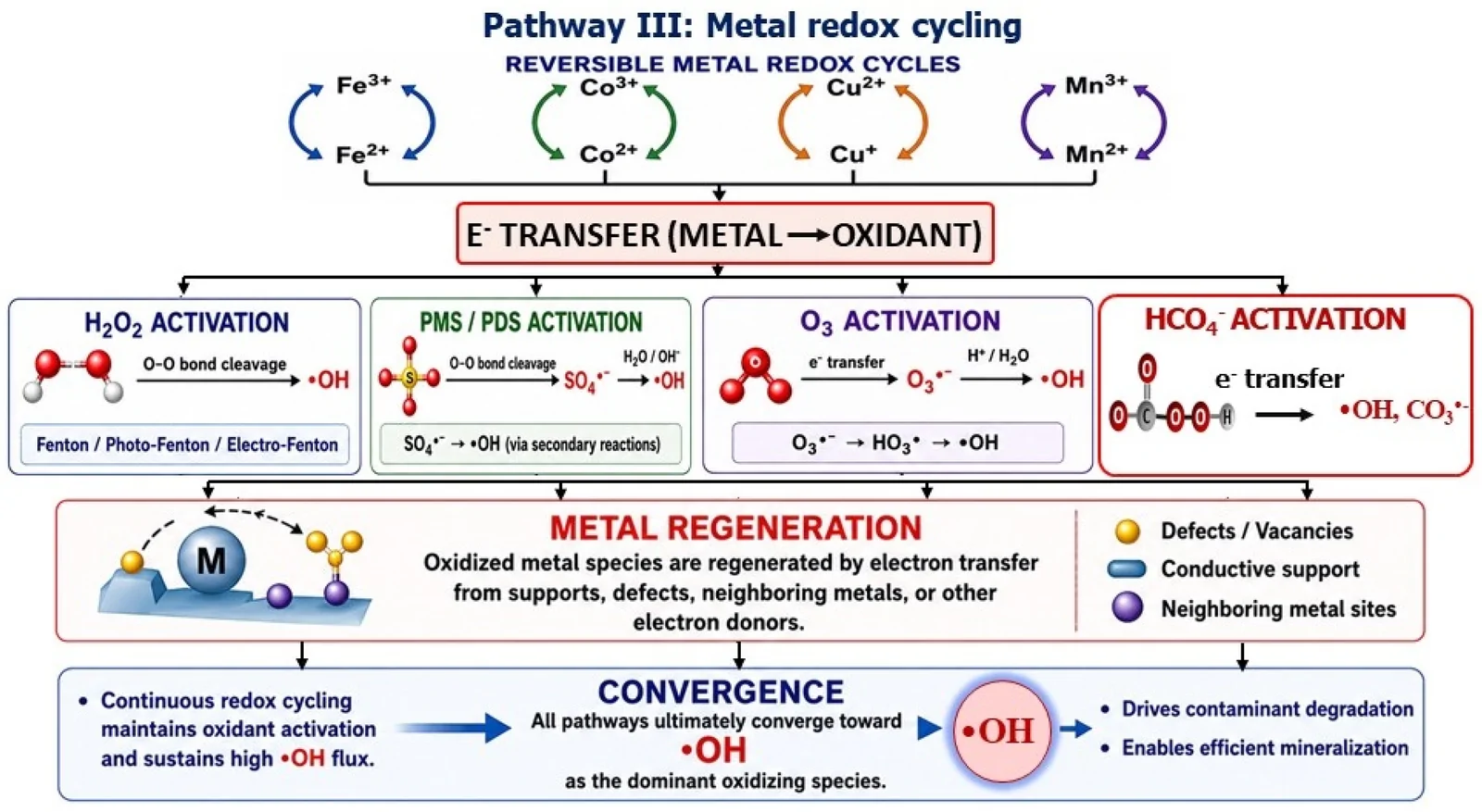
Metal redox cycling as a recurring mechanism for sustained ˙OH generation.

As illustrated in Fig. 8, transition-metal redox couples serve as recyclable electron mediators that continuously transfer electrons to oxidants and regenerate active catalytic sites. Despite differences in oxidant chemistry, electron-transfer-driven activation ultimately sustains a common ˙OH generation network.^121^ Therefore, metal redox cycling can be regarded as an important catalytic amplification mechanism that maintains continuous ˙OH flux across diverse AOP systems.^122^

Overall, metal redox cycling provides an important catalytic pathway that sustains continuous ˙OH generation by coupling electron-transfer processes with oxidant activation, thereby serving as a key mechanistic foundation for many heterogeneous and homogeneous AOP systems.

#### 3.3.2 Fenton, photo-Fenton, and electro-Fenton systems

Fenton-based processes represent one of the most important pathways for ˙OH generation in AOPs. In conventional Fenton systems, Fe^2+^ activates H_2_O_2_ to produce ˙OH according to the classical Fenton reaction (eqn (25)).^10,18^

Because ˙OH is generated directly from H_2_O_2_, the availability and activation efficiency of H_2_O_2_ largely determine oxidation performance.^10,28^ The resulting Fe^3+^ must subsequently be reduced back to Fe^2+^ (eqn (26)), thereby sustaining H_2_O_2_ activation and ˙OH production. In this process, iron functions primarily as a catalytic electron shuttle rather than a direct oxidant source.^28,98^

26 Fe^3+^ + e^−^ → Fe^2+^

Photo-Fenton systems further enhance ˙OH generation by accelerating Fe^3+^ regeneration through photochemical pathways. Under irradiation, ferric hydroxo complexes undergo photoreduction (eqn (27)), while photogenerated electrons from semiconductor photocatalysts continuously convert Fe^3+^ to Fe^2+^.^5–10,106–108^ The accelerated Fe^3+^/Fe^2+^ cycle promotes H_2_O_2_ activation and increases ˙OH production compared with conventional Fenton systems.^30,123,124^

27 FeOH^2+^ + *hν* → ˙OH + Fe^2+^

Electro-Fenton systems enhance ˙OH generation through simultaneous Fe^2+^ regeneration and *in situ* H_2_O_2_ electrogeneration. Cathodic reduction regenerates Fe^2+^ (eqn (26)), while dissolved oxygen undergoes a two-electron oxygen reduction reaction to form H_2_O_2_ (eqn (28)).

28 O_2_ + 2H^+^ + 2e^−^ → H_2_O_2_

**Fig. 9:**
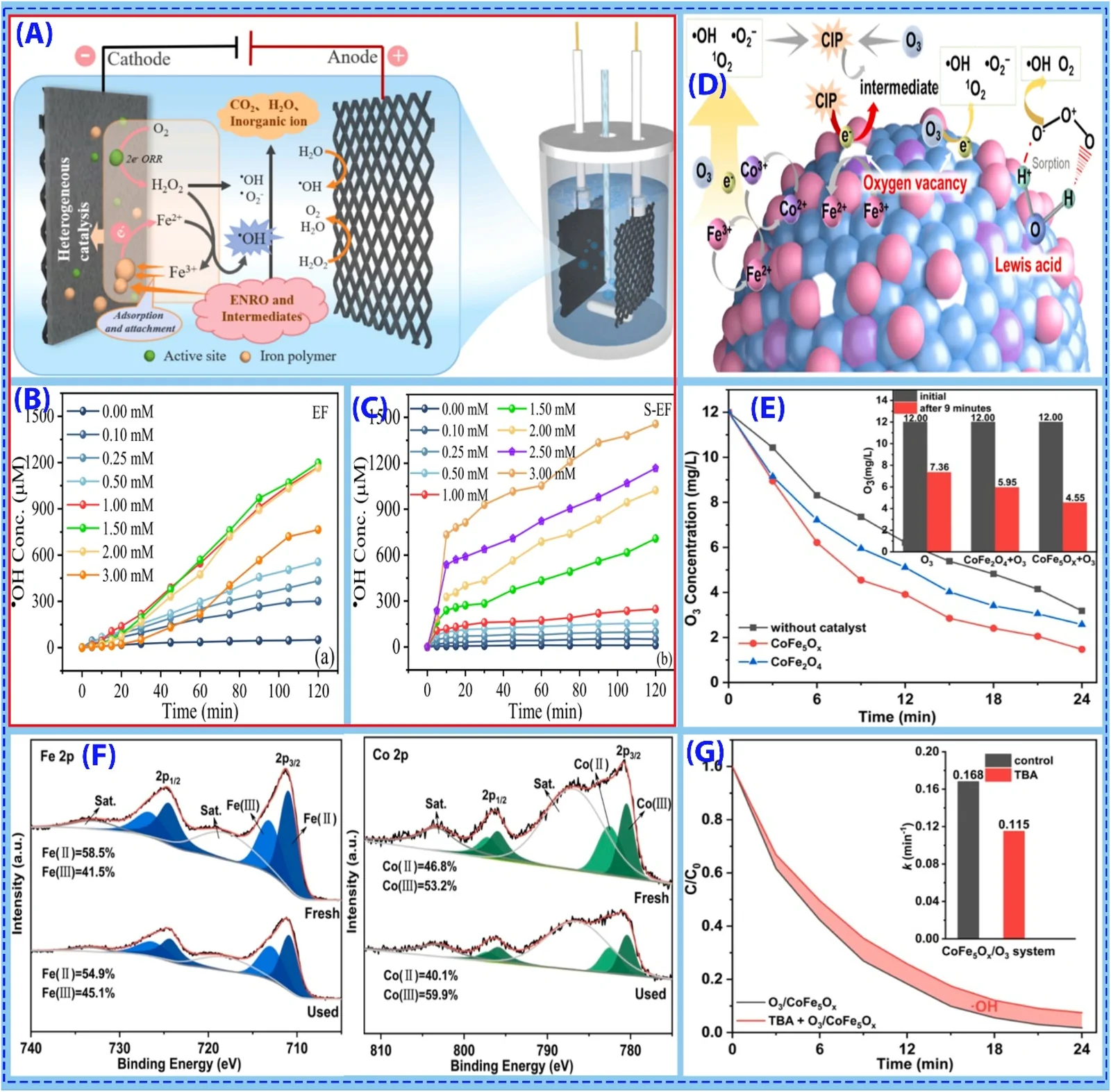
˙OH generation in electro-Fenton and catalytic ozonation systems. (A) Electro-Fenton mechanism.^129^ (B and C) Effect of Fe^2+^ concentration on ˙OH accumulation in conventional and enhanced electro-Fenton systems.^129^ (D) Proposed mechanism of CIP degradation in the CoFe_5_O_*x*_/O_3_ system.^48^ (E) O_3_ decomposition profiles.^48^ (F) XPS spectra of Fe 2p and Co 2p before and after reaction.^48^ (G) Effect of *tert*-butanol (TBA) on CIP degradation, demonstrating the contribution of ˙OH in the CoFe_5_O_*x*_/O_3_ system.^48^ Panels A–C were reproduced with permission from ref. 129, and panels D–G were reproduced with permission from ref. 48. Copyright © Elsevier.

The electrogenerated H_2_O_2_ subsequently reacts with Fe^2+^ to produce ˙OH through the Fenton reaction.^125–128^ As illustrated in Fig. 9A, electro-Fenton systems integrate cathodic H_2_O_2_ production with Fe^3+^/Fe^2+^ redox cycling, establishing a coupled catalytic pathway for ˙OH generation.^129^ A representative study further demonstrated the importance of Fe^3+^/Fe^2+^ cycling for maintaining ˙OH flux. ˙OH accumulation increased with Fe^2+^ concentration up to an optimum level, whereas excess Fe^2+^ reduced net ˙OH availability through scavenging reactions (Fig. 9B).^77,129,130^ In contrast, enhanced Fe^2+^ regeneration enabled sustained ˙OH production over a broader Fe^2+^ concentration range (Fig. 9C), highlighting the critical role of redox cycling in controlling ˙OH generation efficiency.^129,131,132^

Overall, Fenton, photo-Fenton, and electro-Fenton systems share a related ˙OH generation mechanism, in which H_2_O_2_ serves as the direct precursor of ˙OH, while the Fe^3+^/Fe^2+^ redox cycle continuously regenerates active Fe^2+^ sites and sustains oxidant activation.^10,125–128,133^ Therefore, within recurring mechanistic pathway III, metal redox cycling acts as a catalytic amplification mechanism that promotes the persistent conversion of H_2_O_2_ into ˙OH and maintains sustained ˙OH generation during oxidation.

#### 3.3.3 Metal-mediated PMS/PDS and ozone activation

Beyond Fenton chemistry, metal redox cycling can also contribute to the activation of PMS, PDS, and ozone in metal-mediated AOPs. In these systems, transition-metal centers function as recyclable electron shuttles, transferring electrons to oxidants while undergoing reversible valence-state changes. Continuous oxidant activation therefore depends on efficient metal regeneration and electron transport within the catalyst.^16,17,20^

For PMS/PDS systems, reduced metal species such as Co^2+^ and Fe^2+^ donate electrons to peroxide oxidants, initiating oxidant activation and generating sulfate radicals (SO_4_˙^−^):^51,74^

29 Co^2+^ + HSO_5_^−^ → Co^3+^ + SO_4_˙^−^ + OH^−^

30 Fe^2+^ + S_2_O_8_^2−^ → Fe^3+^ + SO_4_˙^−^ + SO_4_^2−^

Because oxidant activation simultaneously oxidizes the metal center, sustained PMS/PDS activation requires continuous regeneration of active species (*e.g.*, Fe^3+^ → Fe^2+^ and Co^3+^ → Co^2+^). The generated SO_4_˙^−^ may subsequently react with water or hydroxide ions to produce ˙OH, thereby linking metal-mediated persulfate activation to ˙OH generation.^16,17,20^

A similar activation mechanism operates during catalytic ozonation.^48,54^ Reduced metal centers transfer electrons to ozone, producing reactive ozone-derived intermediates:

31 Fe^2+^ + O_3_ → Fe^3+^ + O_3_˙^−^

The resulting ozone radical species subsequently undergo decomposition reactions that generate ˙OH:^91,134^32
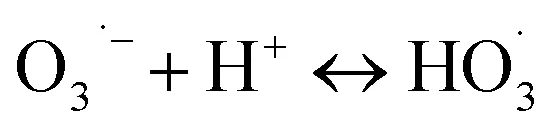
33
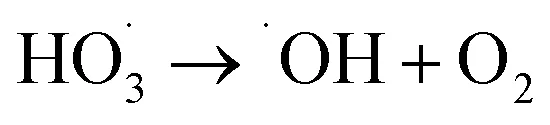


Meanwhile, oxidized metal centers are continuously regenerated through electron-transfer processes. A representative example of metal-redox-mediated ozone activation is shown in Fig. 9D. In the CoFe_5_O_*x*_/O_3_ system, Co^2+^ and Fe^2+^ donate electrons to ozone, promoting its decomposition and subsequent ˙OH formation. The resulting Co^3+^ and Fe^3+^ species are continuously regenerated through Co^3+^/Co^2+^ and Fe^3+^/Fe^2+^ redox cycles, while oxygen vacancies facilitate electron transfer and catalyst reactivation.^48,97^ Experimental results showed enhanced ozone decomposition, oxidation of Co^2+^ and Fe^2+^ to higher valence states (Fig. 9F), and increased ˙OH-dependent degradation (Fig. 9G), supporting the role of metal redox cycling in sustaining ozone activation and ˙OH production.^48,134^

Although PMS/PDS activation and catalytic ozonation involve different primary intermediates (SO_4_˙^−^ and O_3_˙^−^, respectively), both rely on a related mechanistic principle: oxidants act as electron acceptors, whereas transition metals serve as recyclable electron mediators that sustain oxidant activation. Consequently, catalyst characteristics that promote electron mobility and rapid metal regeneration, such as oxygen vacancies, conductive supports, single-atom sites, and multimetallic coupling, can significantly enhance oxidant activation efficiency.^16,17,20,48,81,135^

Overall, PMS/PDS activation and catalytic ozonation exemplify how metal redox cycling functions as an important activation mechanism across different oxidants. While the primary intermediates differ among systems, continuous metal regeneration sustains oxidant activation and ultimately can contribute to ˙OH production during contaminant degradation.

## 4. Cross-system comparison of hydroxyl radical production and utilization

### 4.1. Relative contribution of ˙OH across AOPs

AOPs are often regarded as ˙OH-based technologies because hydroxyl radicals (˙OH) possess an exceptionally high oxidation potential and broad reactivity toward organic contaminants. However, the dependence of different AOPs on ˙OH varies considerably depending on oxidant activation pathways and the associated ROS network. Besides ˙OH, SO_4_˙^−^, superoxide radicals (O_2_˙^−^), singlet oxygen (^1^O_2_), reactive chlorine species (RCS), and ozone-derived oxidants may also contribute significantly to pollutant degradation.^22,24,25,73,74^

**Fig. 10:**
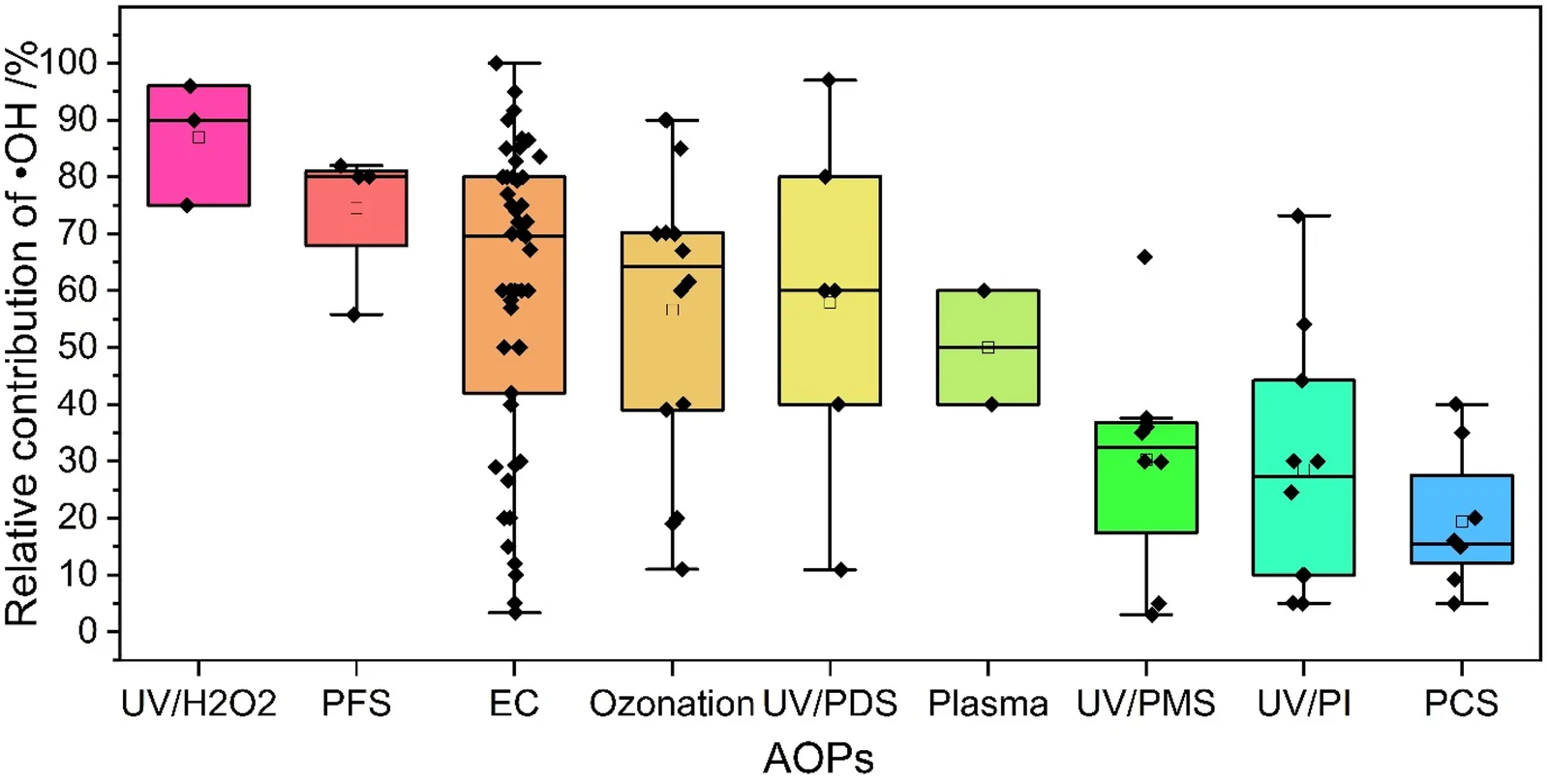
Relative contribution of ˙OH to the degradation of organic pollutants across different AOPs. Notes: PFS = photo-Fenton system; PCS = photocatalysis system; EO = electrochemical oxidation process. Data were compiled and summarized from selected literature sources among ref. 1–151, from which relevant data could be extracted.

Experimental evidence summarized in Table S3 and the comparative distribution illustrated in Fig. 10 demonstrate that the contribution of ˙OH varies markedly among AOP platforms. As shown in Fig. 10, electrochemical oxidation, electro-Fenton, Fenton, photo-Fenton, and UV/H_2_O_2_ systems are generally the most ˙OH-dependent processes, with reported ˙OH contributions frequently exceeding 70–90%.^11–15^ For example, ˙OH contributions of 85.2%, 86.7%, and 91.6% have been reported in representative electrochemical systems.^84,136^ Continuous Fe^3+^/Fe^2+^ cycling further sustains H_2_O_2_ activation and ˙OH regeneration in electro-Fenton processes.^125–128^

Persulfate-based systems (PMS/PDS) represent an intermediate category in which ˙OH and SO_4_˙^−^ radicals commonly coexist. Because SO_4_˙^−^ can be converted into ˙OH through hydrolysis and radical transformation reactions, the relative contribution of ˙OH strongly depends on catalyst composition and operating conditions.^16,17,20,85^ Consequently, PMS/PDS systems are best regarded as co-dominant SO_4_˙^−^/˙OH oxidation systems.

Catalytic ozonation occupies a transitional position between radical and non-radical oxidation pathways. Although ˙OH often dominates under catalytic conditions, direct ozone oxidation can also contribute significantly to pollutant degradation.^21–23,48,89^ In contrast, plasma-based AOPs generate highly complex ROS networks involving ˙OH, O˙, O_3_, O_3_˙^−^, and excited oxygen species, leading to pollutant degradation through synergistic multi-oxidant mechanisms.^24,25,113,114,118^

Overall, the quantitative evidence summarized in Table S3 and Fig. 10 confirms that the role of ˙OH is highly system-dependent and should be evaluated within the context of the overall ROS network rather than assumed *a priori*.^73,74^

### 4.2. ˙OH generation flux *versus* utilization efficiency

˙OH generation is widely used to evaluate AOPs. However, the total amount of ˙OH produced does not necessarily correlate directly with pollutant degradation efficiency. Because ˙OH reactions occur near the diffusion-controlled limit in aqueous systems, ˙OH is highly localized and can undergo recombination or reactions with excess oxidants, catalyst surfaces, or water-matrix constituents before reaching target pollutants.^17,81,137,138^

To distinguish radical production from radical effectiveness, it is useful to differentiate between ˙OH generation flux and ˙OH utilization efficiency. The former describes the rate of ˙OH production, whereas the latter represents the fraction of generated ˙OH that effectively participates in pollutant oxidation:34
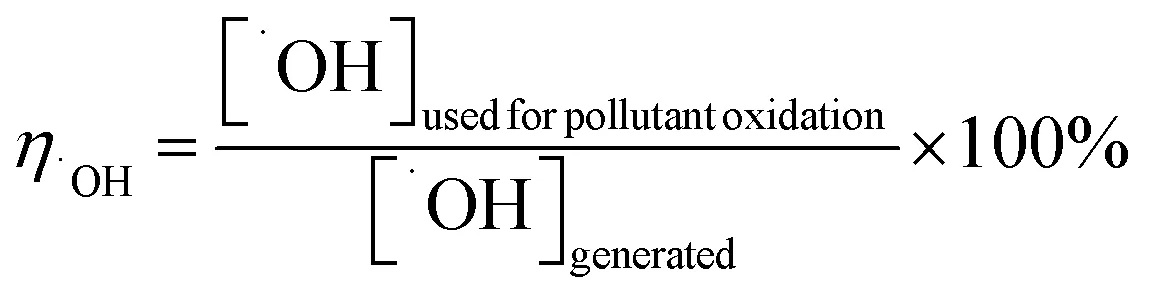
where *η*_˙OH_ denotes the ˙OH utilization efficiency. At present, *η*_˙OH_ should be regarded as a conceptual mechanistic descriptor rather than a standardized performance metric, because independently quantifying ˙OH generation and its productive consumption remains experimentally challenging.

Importantly, no single standardized protocol or benchmark currently exists for determining *η*_˙OH_ across different AOP platforms. Quantitative application would require clearly defined system boundaries, independent estimation of ˙OH generation flux, identification of productive *versus* non-productive radical consumption, and normalization under comparable conditions. Differences in probe chemistry, pollutant concentration, water-matrix composition, reactor configuration, and competing scavenging pathways can further influence the estimated value. Therefore, *η*_˙OH_ should not currently be used as a stand-alone parameter for ranking unrelated AOP technologies, but rather as a conceptual framework for assessing productive ˙OH utilization.

Different AOPs exhibit distinct relationships between radical generation and utilization. UV-based systems can generate high ˙OH fluxes but also experience substantial radical losses through scavenging and side reactions.^8–10^ Electrochemical systems generate ˙OH directly at electrode interfaces, potentially favoring productive oxidation through short diffusion distances.^11–15,100^ Plasma processes can generate extremely high ˙OH fluxes through water dissociation, but simultaneous formation of multiple ROS promotes radical interconversion and recombination.^113,116–118^ Catalytic ozonation occupies an intermediate position, where interfacial coupling between pollutant adsorption and ROS generation can improve radical utilization.^21,22,48,91^

### 4.3. Productive and non-productive pathways of ˙OH in real water matrices

Once generated, ˙OH can undergo either productive or non-productive reaction pathways. Because ˙OH possesses an extremely short lifetime and diffusion distance in aqueous systems, the effectiveness of AOPs depends not only on radical generation but also on the subsequent fate of the generated radicals.^5–8,17^

Productive pathways involve direct reactions between ˙OH and target pollutants through hydrogen abstraction, electron transfer, and electrophilic addition reactions (eqn (35)–(37)). These reactions initiate contaminant transformation, fragmentation, and ultimately mineralization. In this pathway, each generated ˙OH contributes directly to pollutant removal and oxidation efficiency. However, a substantial fraction of ˙OH may be lost through non-productive pathways before encountering pollutant molecules. One important loss route is radical–radical recombination (eqn (38)).^73^

35 ˙OH + RH → R˙ + H_2_O

36 ˙OH + RrH → HO-Ar˙ (Ar: aromatic compound)

37 ˙OH + pollutant → intermediates → CO_2_ + H_2_O

38 ˙OH + ˙OH → H_2_O_2_

Although H_2_O_2_ may subsequently participate in secondary radical-generation reactions, this recombination process reduces the instantaneous concentration of available ˙OH and is generally regarded as a radical-loss pathway.^17,81,139^

**Fig. 11:**
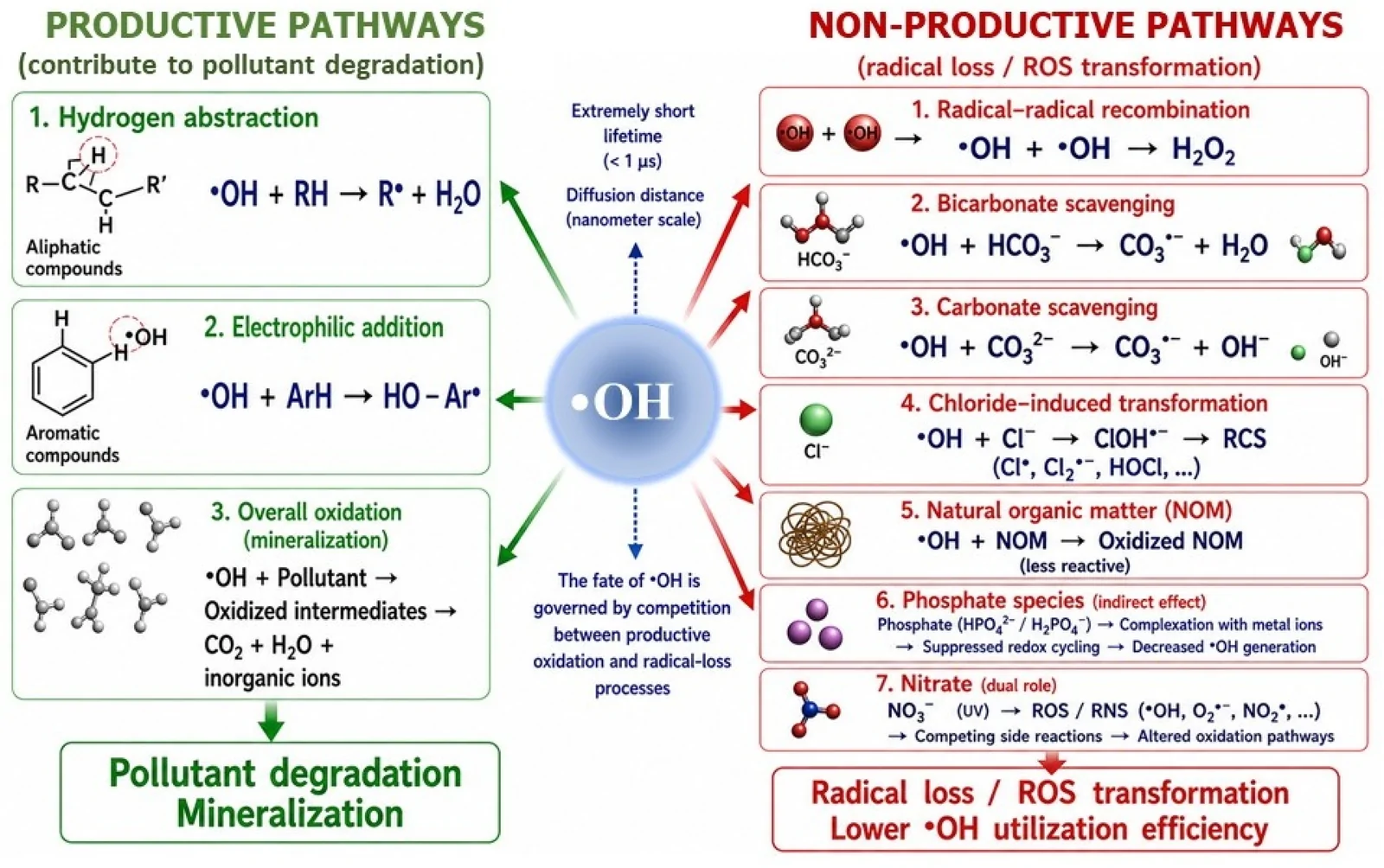
Productive and non-productive pathways of hydroxyl radicals (˙OH) in real water matrices.

Hydroxyl radicals are also readily scavenged by constituents commonly present in natural waters. Bicarbonate and carbonate ions rapidly convert ˙OH into the less reactive carbonate radical (CO_3_˙^−^) (eqn (39) and (40)), while chloride consumes ˙OH and redirects oxidation toward reactive chlorine species (eqn (41)).^69,138,140–142^ Natural organic matter (NOM) is often the most important sink for ˙OH because of its high concentration and strong reactivity toward ˙OH, with reported rate constants typically in the range of 10^8^–10^9^ M^−1^ s^−1^,^7,85,131^ resulting in significant competition with target pollutants. In addition, phosphate species (PO_4_^3−^, HPO_4_^2−^) may indirectly decrease effective ˙OH utilization by suppressing catalytic redox cycles and limiting radical production.^18,122,143^ Phosphate species generally reduce effective ˙OH utilization indirectly by complexing transition-metal ions and suppressing catalytic redox cycles, thereby decreasing radical generation (eqn (42)–(44)).^18,143^ Nitrate (NO_3_^−^) exhibits a dual role: under UV irradiation it may generate additional oxidizing species through photolysis, whereas in other systems it can participate in competing side reactions that alter ROS distribution and pollutant oxidation pathways (eqn (45)–(47)).^28,69,123^ To summarize the competing fates of ˙OH in real water matrices, Fig. 11 illustrates the balance between productive oxidation pathways that contribute directly to pollutant degradation and non-productive scavenging pathways that consume ˙OH without achieving contaminant removal.

39 CO_3_^2−^ + ˙OH → OH^−^ + CO_3_˙^−^

40 HCO_3_^−^ + ˙OH → CO_3_˙^−^ + H_2_O

41 Cl^−^ + ˙OH → ClOH˙^−^

42 Fe^3+^ + PO_4_^3−^ ↔ FePO_4_(s)

43 Fe^3+^ + HPO_4_^2−^ ↔ FeHPO_4_^+^(aq)

44 Co^2+^ + PO_4_^3−^ ↔ Co-PO_4_^−^(complexes)

45
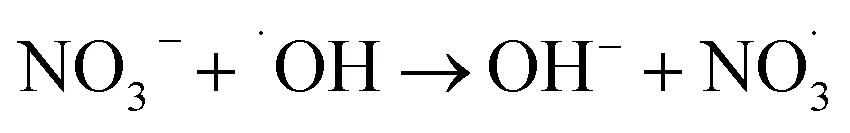
46
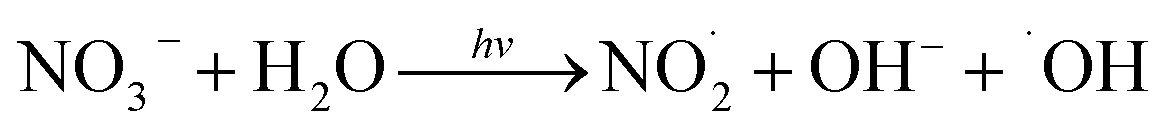
47
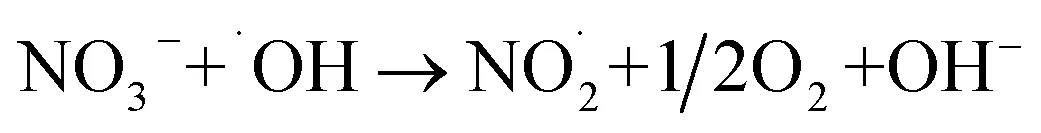


Consequently, the fate of ˙OH is governed by competition between productive pollutant oxidation and non-productive radical-loss processes. The relative importance of these pathways depends strongly on pollutant concentration, catalyst structure, oxidant dosage, and water-matrix composition.^17,81,137,138^ Systems that generate ˙OH in close proximity to adsorbed pollutants can increase the probability of productive pollutant-radical encounters while minimizing radical losses.^99,130^ Therefore, AOP performance is determined not only by how many ˙OH is generated, but also by how many ultimately participate in productive oxidation reactions, providing the mechanistic basis for ˙OH utilization efficiency.^52,131^

### 4.4. Scalability and practical considerations of ˙OH-generating AOPs

Although ˙OH is among the most powerful oxidizing species in AOPs, practical implementation depends not only on radical generation but also on radical fate and overall process requirements. Because ˙OH can be consumed through scavenging, recombination, and side reactions, higher ˙OH flux does not necessarily translate into proportionally higher treatment performance.^131,144^ Thus, scale-up requires balancing effective pollutant oxidation with energy consumption, reagent demand, operational cost, and process stability.

UV-based AOPs (*e.g.*, UV/H_2_O_2_, UV/PDS, UV/PMS, UV/chlorine, and UV/O_3_) provide controllable ˙OH generation and are widely applied in water treatment. However, photon attenuation, oxidant scavenging, and high energy demand can limit effective radical utilization and large-scale deployment.^3,4,145,146^ Fenton and photo-Fenton processes offer high ˙OH yields at relatively low reagent cost, but iron-sludge generation, narrow pH requirements, and sludge management remain major challenges.^10,18,98,147^

Electrochemical and electro-Fenton systems generate ˙OH directly at electrode interfaces, where local pollutant enrichment can favor productive oxidation by reducing radical diffusion losses.^12–15^ However, electricity consumption, electrode cost, and long-term stability remain important barriers.^125,148,149^ Persulfate-based systems (PMS/PDS) offer broad pH applicability and flexible activation pathways, but oxidant cost, incomplete oxidant utilization, and sulfate release can limit large-scale application.^16,17,20,81^ Catalytic ozonation can promote productive pollutant-radical interactions through coupling of ozone decomposition and pollutant adsorption, although ozone generation and gas transfer require substantial energy and infrastructure.^21,22,48,97^ Plasma-based systems can generate high ˙OH fluxes without external oxidants.^25,113,114^ However, simultaneous formation of multiple reactive species can promote radical interconversion and recombination, so high ˙OH production does not necessarily ensure higher treatment efficiency.^116^

Conceptually, lower ˙OH generation may still support effective pollutant oxidation when a larger fraction of generated radicals reacts productively with target contaminants. However, this relationship remains mechanistic rather than quantitative because reliable comparison requires experimentally constrained estimates of radical flux and productive radical utilization. Therefore, ˙OH utilization is considered here as a conceptual design perspective rather than a stand-alone parameter for benchmarking AOP performance or scalability. Overall, scalable ˙OH-based AOPs should promote productive pollutant oxidation while minimizing radical losses, energy demand, reagent consumption, and secondary waste.^52,130,131^ Quantitative assessment of ˙OH utilization across different platforms remains an important direction for future methodological development.

## 5. Catalyst design principles for efficient hydroxyl radical utilization

### 5.1. Promoting productive ˙OH-pollutant interactions

The analysis presented in Section 4 highlights that the effectiveness of ˙OH-based AOPs depends not only on radical generation but also on the fraction of generated radicals that participates in productive pollutant oxidation. Consequently, future catalyst design should move beyond maximizing ˙OH production alone and include strategies that promote productive ˙OH-pollutant interactions.

Improving productive ˙OH utilization requires increasing the probability of pollutant-radical encounters while minimizing non-productive radical-loss pathways. One effective strategy is to promote pollutant adsorption near catalytic active sites, thereby shortening the diffusion distance between pollutants and freshly generated ˙OH and increasing the likelihood of immediate oxidation.^17,81,138^ Interfacial engineering, including hierarchical porosity, surface wettability regulation, defect engineering, and heterostructure construction, can further improve mass transport and strengthen the spatial coupling between pollutant adsorption and radical-generation sites.^16,17,20,81^

In addition, optimizing oxidant activation kinetics, electron-transfer pathways, and active-site distribution can reduce radical self-quenching and secondary scavenging reactions, improving overall radical utilization.^16,17,20,81^ For example, emerging oxidants such as peroxymonocarbonate (HCO_4_^−^) can undergo selective O–O bond cleavage to generate ˙OH and CO_3_˙^−^, while appropriately designed catalysts and activation conditions can favor HCO_4_^−^ activation over competing H_2_O_2_ decomposition.^37,40,93^ Therefore, catalyst design should consider not only oxidant activation efficiency but also activation selectivity and suppression of competing pathways to promote productive ROS formation.

For practical water-treatment applications, catalyst design should also account for water-matrix effects. Preferential pollutant adsorption and selective interfacial oxidation can partially mitigate radical scavenging by bicarbonate, natural organic matter, and other background constituents.^138,142,143^

Therefore, future catalyst development should go beyond radical-generation engineering alone to incorporate radical-fate and oxidant-activation engineering. The design objective is to promote productive ˙OH-pollutant interactions while minimizing non-productive radical-loss pathways under realistic operating conditions, rather than simply maximizing ˙OH production or treating *η*_˙OH_ as a stand-alone optimization target.

### 5.2. Interfacial confinement of ˙OH

Beyond catalyst composition and active-site engineering, controlling the spatial location of ˙OH generation has emerged as an effective strategy for improving AOP performance. Because ˙OH possesses an ultrashort lifetime and nanometer-scale diffusion distance, many radicals generated in bulk solution are lost before reacting with target pollutants.^127,148^ Therefore, the location of radical generation can be as important as the overall radical yield.

Interfacial confinement involves generating ˙OH within localized reaction zones, such as membrane pores, catalyst channels, porous electrodes, and other nanoconfined environments where pollutants are simultaneously transported or enriched.^127,128,148,150^ By spatially coupling radical generation with pollutant accumulation, productive pollutant-radical interactions are enhanced while diffusion-related losses are minimized.

**Fig. 12:**
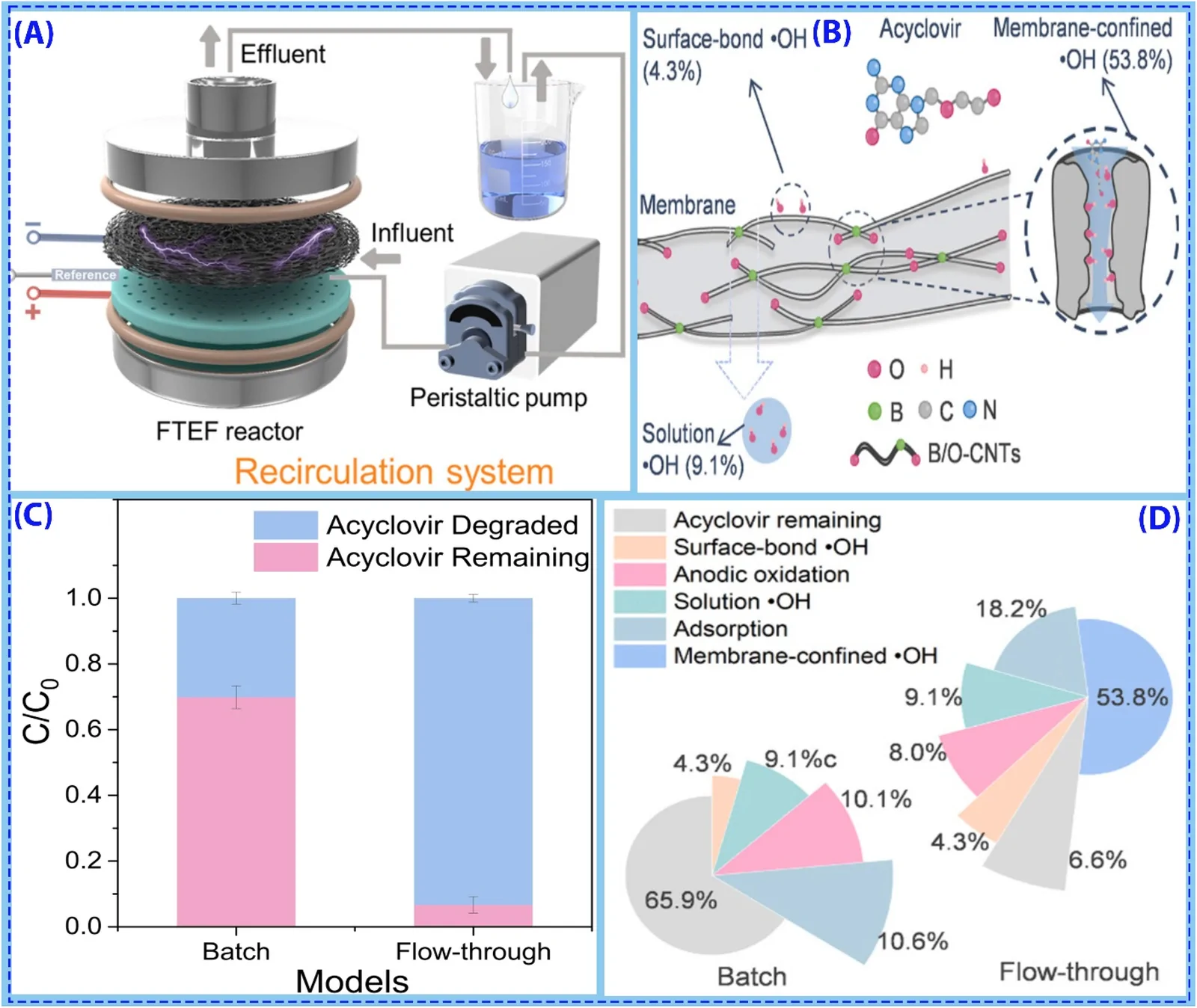
Experimental evidence for interfacial confinement of hydroxyl radicals in a flow-through electrochemical membrane system. (A) Reactor configuration. (B) Schematic representation of acyclovir oxidation by confined and bulk ˙OH species. (C) Comparison of acyclovir removal in batch and flow-through modes. (D) Contributions of different oxidation pathways, demonstrating the predominant role of membrane-confined ˙OH. Panels A–D were reproduced with permission from ref. 127. Copyright © Elsevier.

Experimental evidence supports this concept. As shown in Fig. 12, a flow-through electrochemical membrane reactor achieved substantially higher acyclovir removal than the corresponding batch system.^127^ Mechanistic analysis revealed that membrane-confined ˙OH contributed 53.8% of the overall oxidation activity, whereas dissolved bulk ˙OH accounted for only 9.1% (Fig. 12B, C&D).^127^ These findings demonstrate that oxidation efficiency can be significantly improved by controlling where radicals are generated rather than simply increasing radical production.

This concept provides a mechanistic explanation for the superior performance often observed in membrane electrodes, porous flow-through reactors, and nanoconfined catalytic systems. Therefore, future AOP design should increasingly integrate reaction-location engineering with catalyst engineering, creating confined microenvironments that couple pollutant enrichment, oxidant activation, and localized ˙OH generation to maximize radical utilization under realistic water-treatment conditions.

### 5.3. Local pollutant enrichment around active sites

Beyond strategies that improve radical utilization and reaction-site engineering, increasing pollutant availability at catalytic interfaces represents another important approach for enhancing AOP performance. According to collision-based reaction kinetics, oxidation rates depend not only on oxidant availability but also on substrate concentration. Therefore, increasing pollutant concentration at catalytic interfaces can accelerate oxidation even without increasing ˙OH production.

Local pollutant enrichment refers to the selective accumulation of contaminants near catalytic surfaces through adsorption, partitioning, or specific surface-pollutant interactions. In this framework, catalysts function not only as radical-generation platforms but also as pollutant-concentration media that increase local substrate availability around reactive sites.^33,107^

**Fig. 13:**
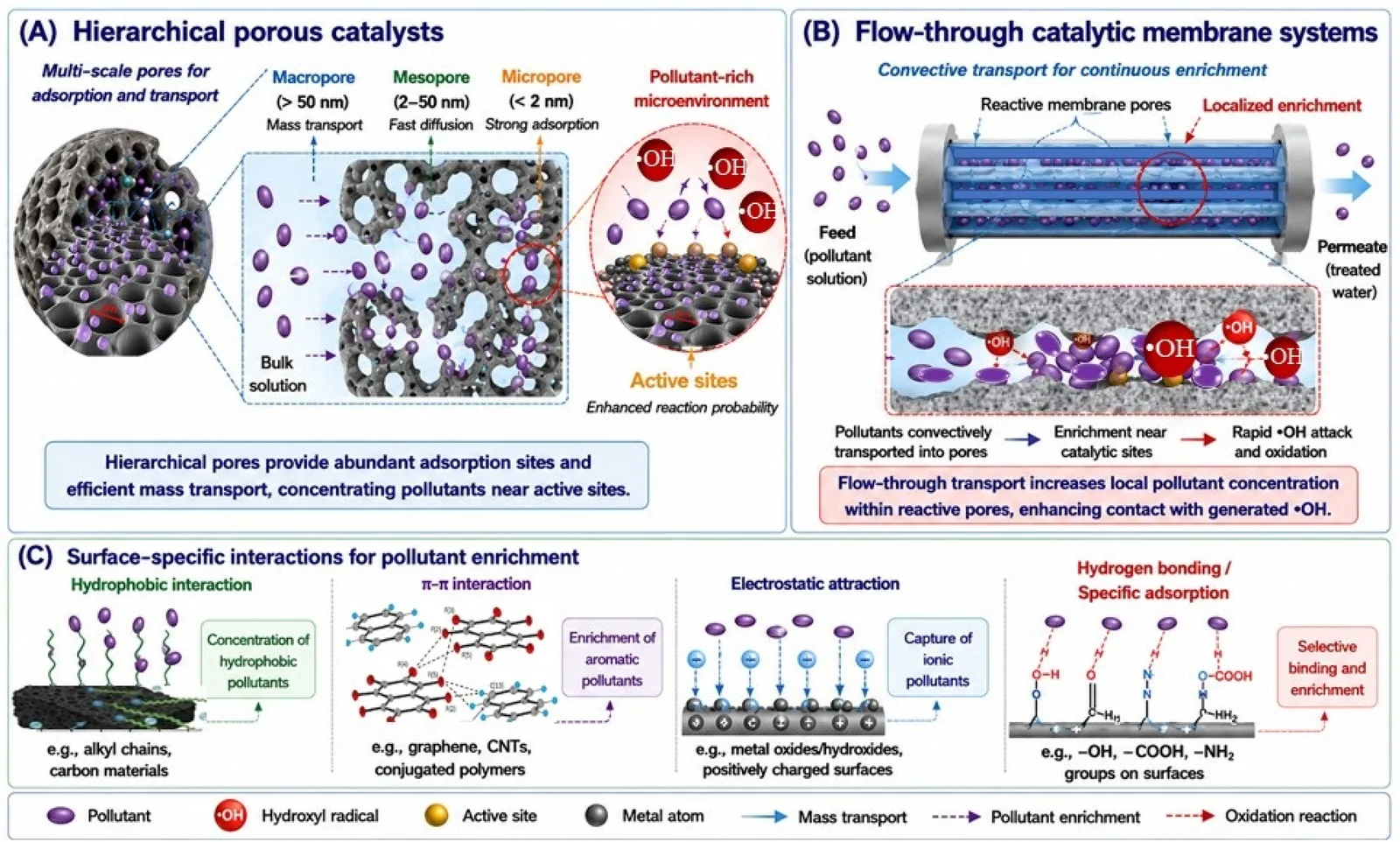
Conceptual illustration of local pollutant enrichment around catalytic active sites. Pollutants can be concentrated near reactive interfaces through (A) hierarchical porous structures, (B) flow-through transport within reactive membrane pores, and (C) surface-specific interactions such as hydrophobic adsorption, electrostatic attraction, and π–π interactions.

Various surface-engineering approaches have been employed to achieve pollutant enrichment. These strategies include tailoring surface chemistry to strengthen pollutant-surface interactions and constructing porous architectures that promote both adsorption and mass transport.^33,107^ Hierarchical porous materials are particularly attractive because their large surface areas and multiscale pore networks provide abundant adsorption sites while maintaining efficient molecular diffusion. As illustrated in Fig. 13, pollutant enrichment can be achieved through a combination of adsorption, selective interfacial interactions, and transport-assisted concentration mechanisms. These approaches increase local substrate availability near active sites and thereby enhance the probability of productive pollutant-oxidant encounters.

This strategy is especially important for trace organic contaminants, whose bulk concentrations are often too low to ensure frequent pollutant-catalyst interactions. By increasing substrate availability at reactive interfaces, adsorption-driven enrichment enhances the probability of oxidation reactions and improves overall treatment efficiency. Therefore, pollutant preconcentration provides a complementary catalyst-design principle that focuses on substrate availability rather than radical production or reaction-location control.

### 5.4. Designing catalysts for real water matrices

Most advanced oxidation catalysts are evaluated using model pollutants in ultrapure water, whereas practical treatment systems contain complex mixtures of inorganic ions, dissolved organic matter, and other background constituents. As a result, catalyst performance observed under laboratory conditions does not necessarily translate directly to real water-treatment applications.^81,137,138,143^

These discrepancies highlight the need to incorporate matrix tolerance into catalyst design and evaluation. Future studies should move beyond idealized testing conditions and systematically assess catalyst performance in the presence of representative water constituents, including bicarbonate, chloride, phosphate, natural organic matter, and mixed-ion systems. Such assessments provide a more realistic measure of catalyst robustness, selectivity, and long-term applicability.^81,95,137,143^

In addition, catalyst stability under prolonged operation becomes increasingly important for practical implementation. Resistance to active-site deactivation, surface fouling, and structural degradation can strongly influence treatment efficiency and operational cost at large scale.

Overall, future catalyst development should emphasize practical applicability alongside catalytic activity. Catalysts that maintain stable performance under realistic water conditions are more likely to achieve successful scale-up and deployment in advanced oxidation technologies.

### 5.5. Mechanism-guided and data-driven ˙OH engineering

The design principles discussed above highlight that effective ˙OH engineering requires simultaneous control of radical generation, productive utilization, interfacial reactions, and pollutant accessibility. Achieving these objectives increasingly relies on mechanism-guided and data-driven approaches capable of identifying the key factors governing ˙OH-based oxidation processes.

Based on the unified framework proposed in Section 3, catalyst performance can be correlated with mechanistic descriptors associated with the three ˙OH-generation pathways, including oxidant adsorption strength, O–O bond activation barriers, electron-transfer kinetics, metal-redox activity, and active-site regeneration efficiency.^16,17,20,81^ Understanding these descriptors can provide a rational basis for predicting catalyst behavior beyond empirical trial-and-error optimization. For emerging oxidants such as HCO_4_^−^, this framework should further incorporate descriptors related to oxidant-activation selectivity, O–O bond cleavage, and competing H_2_O_2_ decomposition, enabling catalyst and activation conditions to be designed toward preferential ROS formation and productive oxidation.

In parallel, pollutant-specific descriptors are increasingly important for understanding oxidation reactivity. Computational studies have shown that parameters such as Fukui indices, HOMO/LUMO distributions, electrostatic potential maps, and bond dissociation energies can identify preferred sites for ˙OH attack and predict degradation pathways.^102,103,150,151^ Linking catalyst properties with pollutant-reactivity descriptors may therefore facilitate more efficient catalyst-pollutant matching for specific treatment applications.

**Fig. 14:**
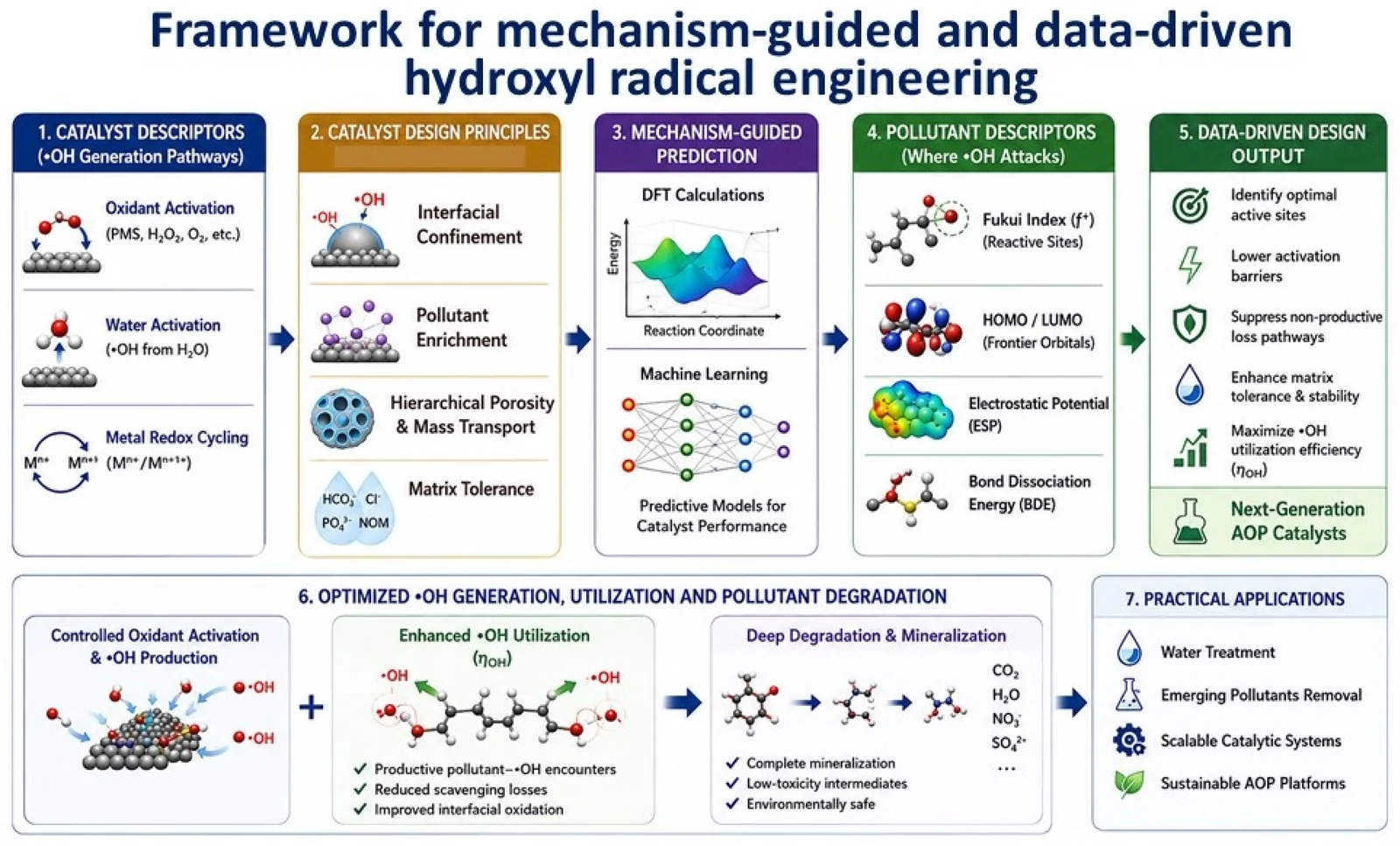
Framework for mechanism-guided and data-driven ˙OH engineering.

The integration of density functional theory (DFT), *operando* characterization, high-throughput screening, and machine-learning tools provides new opportunities for predictive catalyst development. Fig. 14 summarizes a mechanism-guided framework in which catalyst descriptors governing ˙OH generation are integrated with catalyst-design principles, pollutant-reactivity descriptors, and data-driven prediction tools to guide catalyst development. By combining mechanistic understanding with predictive modeling, future studies may accelerate the discovery of catalysts that promote productive ˙OH utilization and pollutant oxidation under realistic operating conditions, while providing experimentally testable hypotheses for controlling radical fate and interfacial reactivity.

## 6. Conclusion

Hydroxyl radicals are central reactive species in AOPs, yet diverse oxidants, catalysts, and activation technologies have led to fragmented mechanistic interpretations. This review establishes a unified ˙OH-centered framework connecting diverse AOPs through common reaction principles.

For the AOP systems considered in this review, ˙OH generation can be organized into three recurring mechanistic routes: (i) oxidant activation, (ii) generation from water, and (iii) metal redox cycling. These pathways can contribute to interconnected ˙OH reaction networks, providing a unified perspective for understanding ROS evolution and contaminant degradation. Importantly, AOP performance depends not only on ˙OH generation but also on radical fate and productive pollutant oxidation. Accordingly, *η*_˙OH_ is proposed as a conceptual mechanistic descriptor rather than a standardized metric for direct performance benchmarking. Key catalyst-design principles therefore include promoting productive ˙OH-pollutant interactions, interfacial confinement, local pollutant enrichment, balanced radical/non-radical pathways, and tolerance to complex water matrices.

Future advances will benefit from mechanism-guided control of ˙OH generation, reaction location, radical fate, and pollutant accessibility. Quantitative investigation of ˙OH flux and utilization, combined with *operando* characterization, theoretical modeling, and machine-learning-assisted design, may enable more efficient and selective AOP systems under realistic conditions. Overall, this review provides a conceptual foundation for mechanism-guided catalyst design and future quantitative investigation of radical utilization.

## Author contributions

Hoang Hien Y: investigation, resources, methodology, writing – original draft. Nguyen Thi Thy Nga, Bui Dinh Nhi, Minh Thi Thao, Hai Son Truong-Lam, Ha Huu Do: conceptualization, resources, investigation, methodology. Nguyen Tien Hoang: writing – review & editing, writing – original draft, methodology, investigation, formal analysis, data curation.

## Conflicts of interest

The authors declare that they have no known competing financial interests or personal relationships that could have appeared to influence the work reported in this paper.

## Supplementary Material

RA-OLF-D6RA06527A-s001

## Data Availability

The data underlying Fig. 10 were compiled and summarized from selected literature sources cited in the manuscript (ref. 1–151). The corresponding source references are provided in the manuscript. No new experimental data were generated in this review. Supplementary information (SI) is available. See DOI: https://doi.org/10.1039/d6ra06527a.
