# Supplementary material for: Hydroxyl radical chemistry in advanced oxidation processes: a cross-system mechanistic framework and catalyst design perspectives
Source: RSC Adv. 2026 Sep 23;16(44):54512–37. doi: 10.1039/d6ra06527a (PMC13599134; doi:10.1039/d6ra06527a)
Supplement: Supplementary file 1 [file RA-016-D6RA06527A-s001.pdf]

Supplementary Information (SI) for RSC Advances.
This journal is © The Royal Society of Chemistry 2026

Supporting formation for

Hydroxyl Radical Chemistry in Advanced Oxidation Processes: A Unified Cross-System Mechanistic Framework and Catalyst Design Perspectives

Hoang Hien Y[1,2a], Nguyen Tien Hoang[3a*], Nguyen Thi Thy Nga[3], Bui Dinh Nhi[4], Minh Thi Thao[5], Hai Son Truong-Lam[6], Ha Huu Do[7,8]

[1]Center for Advanced Chemistry, Institute of Research & Development, Duy Tan University, 03 Quang Trung, Da Nang City, 550000, Vietnam

[2]Faculty of Environmental and Natural Sciences, Duy Tan University, 03 Quang Trung, Da Nang City, 550000, Vietnam.

[3]The University of Danang, University of Science and Education, 459 Ton Duc Thang st., Da Nang 550000, Viet Nam

[4]Faculty of Applied Science and Engineering, Electric Power University, Ha Noi, Viet Nam

[5]Faculty of Energy and Sustainable Development, Electric Power University, Ha Noi, Viet Nam

[6]Faculty of Chemistry, University of Science, Vietnam National University Ho Chi Minh City, 70000, Viet Nam

[7]NTT Hi-Tech Institute, Nguyen Tat Thanh University, Ho Chi Minh City, Vietnam

[8]Nguyen Tat Thanh University Center for Hi-Tech Development, Saigon Hi-Tech Park, Ho Chi Minh City, Vietnam

Table S1. Comparison of •OH with other free radicals [1]

Reactivity	•OH	$SO_4^{\bullet-}$	$Cl^{\bullet-}$	$^1O_2$	$\bullet O_2^-$

* Corresponding author at: The University of Danang, University of Science and Education, 459 Ton Duc Thang st., Da Nang 550000, Viet Nam. Email: nthoang@ued.udn.vn.
[a]The authors contributed equally.

species					
Oxidation potential (V)	2.8	2.5 – 3.1	2.4 – 2.5	1.07	2.4
Half-life (µs)	< 1	30 - 40	~ (a few) µs; $\sim 10^{-6}$–10^{-5} s.	3 – 4.2	-
Selectivity with pollutant	None-selectivity [2].	Electron-donating pollutants [2].	Electrophilic selectivity	Electron-donating pollutants	Poor selectivity

Table S2. Reaction rate constants of •OH compared to other free radicals

Organic Compounds	•OH, 10^9	$SO_4^{\bullet-}$, 10^9	$Cl^{\bullet-}$, 10^9	$^1O_2$, 10^6	$\bullet O_2^-$, 10^6
	(M^{-1} s^{-1})				
**GROUP 1: Phenol & Chlorophenol**					
Phenol	6.6	1.3 – 1.8	1	1	0.002
4-Chlorophenol	9.3	2.5	2	0.5	0.005
2,4-Dichlorophenol	4.3	7.4	3	0.3	0.1
2,4,6-Trichlorophenol	2	0.2	4	0.1	0.8
**GROUP 2: Bisphenol & Parabens**					
Bisphenol A	6.9 – 8.5	1.4	1.5	0.8	0.001
Methylparaben	5	1.2	0.5	0.8	0.001
Ethylparaben	5.2	1.3	0.55	5	0.01
Propylparaben	5.4	1.35	0.6	0.8	0.01
Butylparaben	5.6	1.4	0.65	0.8	0.01
**GROUP 3: Fluoroquinolones antibiotics**					
Ciprofloxacin	6	1	1	0.01	0.1
Norfloxacin	1	0.8	0.8	0.01	0.1
Ofloxacin	6	1	1	0.01	0.1
Levofloxacin	5.5	0.9	0.9	0.01	0.1
Enrofloxacin	6	1	1	0.01	0.1
**GROUP 4: Sulfonamides and other antibiotics**					
Sulfadiazine	2	0.8	0.4	0.05	0.001
Sulfamethoxazole	5.82	2.98	0.5	0.08	0.001

Sulfathiazole	4	0.6	0.3	0.05	0.001
Trimethoprim	6.5	0.8	0.5	0.05	0.001
**GROUP 5: Pesticides and other aromatic compounds**					
Atrazine	2.3	2.6	0.5	0.01	0.001
Carbamazepine	8	1	0.6	0.05	0.001
Diclofenac	8.4	1	0.6	1	0.001
Triclosan	12	3	2	0.5	0.001

Notes: Reaction rate constants were compiled from authoritative kinetic databases and experimental studies using pulse radiolysis, laser flash photolysis, and relative rate methods. Hydroxyl radical data were primarily obtained from [3,4]. Sulfate, chlorine, singlet oxygen, and superoxide radical kinetics were referenced from [5-9]. Reported values represent typical ranges under environmentally relevant aqueous conditions.

Table S3. Role and contribution ratio of •OH radicals in electrolytic catalytic systems.

Electrolytic / Catalytic System	Electrode / Catalyst Material	Dominant Free Radicals	Fraction / Concentration / Contribution	Ref.
AOPs – PFOA	Anodic oxidation	•OH	Dominant reaction, k = 5.05×10^9 $M^{-1}s^{-1}$	10
E-nZVC/BC-PMS	nZVC/BC	•OH, $^1O_2$, $SO_4^{•-}$	•OH, $^1O_2$ major role; $SO_4^{•-}$ secondary role	11
ENR degradation	-	•OH, Cl•/$Cl_2^{•-}$	•OH: 85.15 % (pH 8)	12
PS reduction	Cathodic PS reduction	$SO_4^{•-}$ → •OH	•OH is primarily generated in acidic condition	13
AR14 oxidation	-	•OH	86.7 %, 76.4 %, 82.8 % (after 6–20 minutes)	14
m-NP oxidation	PbO_2–zeolite	•OH	•OH dominates, increasing with current density and pH	15
E-PMS catalysis	-	•OH	Dominant role; reaction rate significantly decreases with TBA addition	16
Pt@MOF(Fe)/NF	Ni foam	•OH	•OH is the primary radical for PDS activation	17
ECMR–O_3	ECMR–O_3	•OH	58.3 % contribution to COD removal	18
SA–Co–C_3N_5–EF	SA–Co–C_3N_5	•OH	892.3 μM, highest •OH concentration	19

UVC/e-H_2O_2@e-ClO^-	-	•OH, Cl•	•OH is dominant, with synergistic effects increasing TOC removal by 39.8 %	20
CBZ oxidation	-	•OH, $SO_4^{•-}$	•OH: 15.7 % (initial stage)	21
PFAAs oxidation	-	•OH	•OH: 69.5 %, the primary radical responsible for Co–EDTA degradation	22
Ce-promoted system	-	•OH	The steady-state concentration of •OH increases markedly with Ce and applied voltage	23
CuMn–Sb–SnO_2	Cu, Mn-doped SnO_2 anode	•OH	Dominant role, strongly enhanced by Cu and Mn	24
3D-MoS_2/PDS	3D catalytic system	•OH	Strong EPR signal, the primary radical	25
CuFe–Sb–SnO_2	CuFe–Sb co- doped SnO_2	•OH	91.63 % contribution	26
UV–BDD	BDD	•OH (surface), •OH (free)	•OH (surface) is dominant; UV irradiation generates additional •OH (free)	27
EO-PMS	-	•OH, $SO_4^{•-}$	Two major oxidizing radicals	28
Flow-membrane	Flow-through membrane	•OH (membrane, sol., surface)	53.8 %, 9.1 %, 4.3 %	29
p-SiC/BDD-1.2 %	BDD-SiC	•OH	72.1 % contribution, exceeding $^1O_2$ and $SO_4^{•-}$	30
EA-FeNiO/GF-PS	GF-PS	•OH, $SO_4^{•-}$	•OH: 26.6 %; $SO_4^{•-}$: 38.1 %	31
Fe-EC/S(IV)	-	•OH	A secondary but important role	32
3DER	CeO_2/Fe_3O_4/NiFe-LDH	•OH	Significantly higher •OH generation	33
Co/Ce–C350Air	Co/Ce composite	•OH	Dominant surface-bound radical	34
Solar/N–TiO_2/EAOPs	N–TiO_2	•OH, Cl•	•OH ≈ 77 % contribution	35
Electro-chlorination	-	•OH, $Cl_2^{•-}$	Cl^- inhibits •OH, reducing its concentration from 3.3 to 0.95 × 10^{-13} M	36
CoNi/PILs-rGO	Cathode–H_2O_2	•OH	High •OH generation,	37

		confirmed by TBA scavenging

References

1. Li C, Chen G, Guo J, Shi Y, Li J, Tang J. FeS/rGO modified cathode for enhanced electrochemical activity and TCE degradation in PS-MFC system. *J Environ Chem Eng*. 2025;13(3):116464. doi:10.1016/j.jece.2025.116464

2. Fu Q, Wang XX, He H, et al. A comparative study on the degradation of carbamazepine by UV laser/chlorine and UV laser/PS processes: Performance and mechanisms. *Chem Eng J*. 2024;484(January). doi:10.1016/j.cej.2024.149496

3. Buxton G V., Greenstock CL, Helman WP, Ross AB. Critical Review of rate constants for reactions of hydrated electrons, hydrogen atoms and hydroxyl radicals (·OH/·O − in Aqueous Solution. *J Phys Chem Ref Data*. 1988;17(2):513-886. doi:10.1063/1.555805

4. Haag WR, Yao CCD. Rate constants for reaction of hydroxyl radicals with several drinking water contaminants. *Environ Sci Technol*. 1992;26(5):1005-1013. doi:10.1021/es00029a021

5. P. Neta, R. E. Huie ABR. Rate constants for reactions of inorganic radicals in aqueous solution. *J Phys Chem Ref Data*. 1988;17(3):1027-1284.

6. Wojnárovits L, Wang J, Chu L, Takács E. Rate constants of chlorine atom reactions with organic molecules in aqueous solutions, an overview. *Environ Sci Pollut Res*. 2022;29(37):55492-55513. doi:10.1007/s11356-022-20807-9

7. Wojnárovits L, Takács E. Rate constants of dichloride radical anion reactions with molecules of environmental interest in aqueous solution: a review. *Environ Sci Pollut Res*. 2021;28(31):41552-41575. doi:10.1007/s11356-021-14453-w

8. Wilkinson F, Helman WP, Ross AB. Rate Constants for the Decay and Reactions of the Lowest Electronically Excited Singlet State of Molecular Oxygen in Solution. An Expanded and Revised Compilation. *J Phys Chem Ref Data*. 1995;24(2):663-677. doi:10.1063/1.555965

9. Bielski BHJ, Cabelli DE, Arudi RL, Ross AB. Reactivity of HO2/O−2 Radicals in Aqueous Solution. *J Phys Chem Ref Data*. 1985;14(4):1041-1100. doi:10.1063/1.555739

10. Das A, Adak A. Electrochemical oxidation of perfluorooctanoic acid (PFOA) using graphite substrate lead dioxide (GSLD) as anode: Investigating reaction mechanism, degradation byproducts and influences of process parameters. *J Environ Chem Eng*. 2025;13(3):116869. doi:10.1016/j.jece.2025.116869

11. Zhang W, Bi J, Hui Y, Wang G, Ma R, Wang H. Enhanced ofloxacin degradation by three-dimensional electrochemical activation of peroxymonosulfate using nano ZVC doped reed straw biochar particle electrode. *J Environ Chem Eng*. 2025;13(6):119512. doi:10.1016/j.jece.2025.119512

12. Chen J, Yang HM, Feng Y, Liu L, Gao YQ, Shang X. The electrochemical degradation of enrofloxacin using RuO2-IrO2-TiO2/Ti electrodes: Kinetics, mechanism, and model prediction. *J Water Process Eng*. 2025;72(January). doi:10.1016/j.jwpe.2025.107519

13. Cerrón-Calle GA, Flores M, Roldan MA, et al. Electrochemical persulfate activation for

degradation of BPA using a novel self-standing CuOx/CoOx cathode. *Chem Eng J*. 2025;519(February). doi:10.1016/j.cej.2025.164960

14. Wu T, Hu Z, Yang J, et al. Insight into the roles of Cl− for the degradation of Acid Red 14 in an electrochemical advanced oxidation system: Mechanisms and DFT studies. *Chem Eng J*. 2025;503(November 2024):158079. doi:10.1016/j.cej.2024.158079

15. Duan X, Li Y, Sui X, Zhao X, Ren X, Chang L. Electrochemical degradation of m-nitrophenol using an innovative zeolite modified PbO2 anode: Electrode characterization, influence factors and degradation mechanism. *J Environ Chem Eng*. 2024;12(5):113682. doi:10.1016/j.jece.2024.113682

16. Liu Z, Bikogiannakis AK, Lynen F, et al. Degradation of ciprofloxacin by electrochemically assisted peroxydisulfate activation with core-shell CoFe2O4/CoFe@C particle electrodes and RVC cathode: Synergistic performance, environmental applicability, and toxicity evaluation. *Sep Purif Technol*. 2025;376(May). doi:10.1016/j.seppur.2025.133817

17. Zhang F, Zhang X, Han P, Cui J, Wang C, Chen Q. Electrochemical activation of persulfate for degradation of tetracycline hydrochloride based on Pt@MOF(Fe)/NF cathode. *J Environ Chem Eng*. 2025;13(2):115966. doi:10.1016/j.jece.2025.115966

18. Lv T, Du M, Wang R, et al. An integrated process of electrochemical membrane reactor coupled with O3 and aerobic biological treatment for organic removal from soil leachate during contaminated soil remediation. *Sep Purif Technol*. 2025;379(P2):134985. doi:10.1016/j.seppur.2025.134985

19. Li Y, Yan M, Lin H, Wang S, Duan X, Wu XL. Electrochemical generation of H*-•OH redox pair for rapid removal of refractory nitro-organic micropollutants. *Appl Catal B Environ*. 2025;361(July 2024). doi:10.1016/j.apcatb.2024.124663

20. Souto RS, Sánchez-Montes I, Santos GOS, et al. Electrochemical degradation of tebuthiuron pesticide using a flow reactor: Insights into the co-generation of oxidants and ecotoxicological risks. *J Environ Chem Eng*. 2025;13(2). doi:10.1016/j.jece.2025.115528

21. Liu Z, Liu R, Chen S, et al. Catalytic Peroxymonosulfate activation via In-Situ ketone Derivatives: Unveiling dual kinetics in electrochemical degradation. *Sep Purif Technol*. 2025;375(April):133680. doi:10.1016/j.seppur.2025.133680

22. Li M, Zhang H, Cheng J, et al. Unveiling the decomposing and mineralizing mechanism of novel perfluoroalkyl acids via hydroxyl radical dominated electrochemical oxidation. *Appl Catal B Environ*. 2024;351(January):123983. doi:10.1016/j.apcatb.2024.123983

23. Wu JC, Tsai CW, Hou CH. Unveiling species transformation in electrochemical synergy of Co-EDTA decomplexation and cobalt recovery. *Chem Eng J*. 2025;521(July):167014. doi:10.1016/j.cej.2025.167014

24. Chen Y, Liu C, Wang C, et al. Cu/Mn-mediated electron shuttle and high-valent metals enhance hydroxyl radicals production during the electrochemical oxidation on the CuMn-Sb-SnO2 electrode. *J Hazard Mater*. 2024;477(July):135288. doi:10.1016/j.jhazmat.2024.135288

25. Li X, Zuo S, You B, et al. Enhancing carbamazepine degradation in electrochemical

systems: The interplay of sulfur vacancies and absorbed peroxydisulfate in molybdenite electrodes. *Sep Purif Technol*. 2025;358(PB):130431. doi:10.1016/j.seppur.2024.130431

26. Chen Y, Tang H, Sun H, Liu Y, Xie H, Wang L. A novel CuFe-Sb-SnO2 anode for efficient degradation of ciprofloxacin by enhancing hydroxyl radical production: Key role of Cu/Fe in accelerating electron transfer. *Sep Purif Technol*. 2025;356(PB):129996. doi:10.1016/j.seppur.2024.129996

27. Chen P, Mu Y, Chen Y, et al. Shifts of surface-bound •OH to homogeneous •OH in BDD electrochemical system via UV irradiation for enhanced degradation of hydrophilic aromatic compounds. *Chemosphere*. 2022;291(November 2021). doi:10.1016/j.chemosphere.2021.132817

28. Abdelrahman A, Ganiyu SO, Gamal El-Din M. Degradation of surrogate and real naphthenic acids from simulated and real oil sands process water using electrochemically activated peroxymonosulfate (EO-PMS) process. *Sep Purif Technol*. 2023;306(PA):122462. doi:10.1016/j.seppur.2022.122462

29. Chen J, Zhang Q, Zhong S, et al. Bifunctional flow-through electrified-membrane for intensified confined hydroxyl radical generation and efficient pollutant degradation. *Chem Eng J*. 2025;523(July):168656. doi:10.1016/j.cej.2025.168656

30. Wang X, Dou J, Deng Z, et al. Three-dimensional porous SiC/BDD electrode with long-term robustness and enhanced electrochemical degradation performance for refractory organic pollutants. *Chem Eng J*. 2024;499(October). doi:10.1016/j.cej.2024.156454

31. Teng X, Wang Z, Abukhadra MR, Khaled Mahmoud R, Qu R. Promoted electrochemical degradation of triclosan with persulfate on Fe-Ni oxide modified graphite felt cathode. *Sep Purif Technol*. 2024;344(March):127229. doi:10.1016/j.seppur.2024.127229

32. Zhu J, Ding W, Jiang Z, Peng T, Tan X. Electrochemically sustained iron-activated sulfite process for rapid contaminant degradation and intrinsic bromate suppression at near-neutral pH. *J Environ Chem Eng*. 2025;13(5):118548. doi:10.1016/j.jece.2025.118548

33. Zhang H, Sun F, Zhang F, et al. Rationally designed CeO2/Fe3O4/NiFe-LDH particle electrodes facilitate hydroxyl radical generation for the degradation of N-nitrosodimethylamine. *Sep Purif Technol*. 2025;377(P3):134471. doi:10.1016/j.seppur.2025.134471

34. Xie T, Wang L, Wang H, Cao C, Tang C, Hu X. In-situ electrodeposition of Co/Ce-MOF-derived carbon composites as anode for electrochemical degradation of ceftazidime. *Sep Purif Technol*. 2024;330(PA):125231. doi:10.1016/j.seppur.2023.125231

35. Bai J, Zhao T, Han G, Wang Y, Wang L, Wu X. Degradation of ibuprofen in a solar-activated, electrically produced hypochlorite system catalyzed by N-TiO2: Its synergistic mechanism, free radical cycling, and microbial effects. *J Environ Chem Eng*. 2025;13(5):118419. doi:10.1016/j.jece.2025.118419

36. Yuan J, Li Y, Chen X, Yi Q, Wang Z. One electron oxidation-induced degradation of brominated flame retardants in electroactive membrane filtration system: Vital role of dichlorine radical-mediated process. *J Hazard Mater*. 2024;471(April):134318.

doi:10.1016/j.jhazmat.2024.134318

37. Chen C, Bai S, Liu P, et al. Boosted electrochemical reduction detoxification and oxidation of clofibric acid by polymerized ionic liquid functionalized cathode: Parameter optimization and degradation mechanisms. *J Environ Chem Eng*. 2024;12(6):114477. doi:10.1016/j.jece.2024.114477
